# The relationship between seasonal influenza and telephone triage for fever: A population-based study in Osaka, Japan

**DOI:** 10.1371/journal.pone.0236560

**Published:** 2020-08-06

**Authors:** Yusuke Katayama, Kosuke Kiyohara, Sho Komukai, Tetsuhisa Kitamura, Kenichiro Ishida, Tomoya Hirose, Tasuku Matsuyama, Takeyuki Kiguchi, Atsushi Hirayama, Takeshi Shimazu

**Affiliations:** 1 Department of Traumatology and Acute Critical Medicine, Osaka University Graduate School of Medicine, Suita, Japan; 2 Department of Food Science, Faculty of Home Economics, Otsuma Women’s University, Tokyo, Japan; 3 Division of Biomedical Statistics, Department of Integrated Medicine, Graduate School of Medicine, Osaka University, Suita, Japan; 4 Division of Environmental Medicine and Population Sciences, Department of Social and Environmental Medicine, Osaka University Graduate School of Medicine, Suita, Japan; 5 Department of Acute Medicine and Critical Care Medical Center, Osaka National Hospital, National Hospital Organization, Osaka, Japan; 6 Emergency and Critical Care Center, Osaka Police Hospital, Osaka, Japan; 7 Department of Emergency Medicine, Kyoto Prefectural University of Medicine, Kyoto, Japan; 8 Kyoto University Health Services, Kyoto, Japan; INSERM, FRANCE

## Abstract

**Background:**

Replacing traditional surveillance with syndromic surveillance is one of the major interests in public health. However, it is unclear whether the number of influenza patients is associated with the number of telephone triages in Japan.

**Methods:**

This retrospective, observational study was conducted over the six-year period between January 2012 to December 2017. We used the dataset of a telephone triage service in Osaka, Japan and the data on influenza patients published from the Information Center of Infectious Disease in Osaka prefecture. Using a linear regression model, we calculated Spearman’s rank-order coefficient and R^2^ of the regression model to assess the relationship between the number of telephone triages for fever and the number of influenza patients in Osaka. Furthermore, we calculated Spearman’s rank-order coefficient and R^2^ between the predicted weekly number of influenza patients from the linear regression model and the actual weekly number of influenza patients for influenza outbreak season (December-April).

**Results:**

There were 465,971 patients with influenza, and the number of telephone triages for fever was 420,928 among 1,065,628 total telephone triages during the study period. Our analysis showed that the Spearman rank-order coefficient was 0.932, and R^2^ and adjusted R^2^ were 0.869 and 0.842, respectively. The Spearman rank-order coefficient was 0.923 (P<0.001) and R^2^ was 0.832 in December-April (*P*<0.001).

**Conclusion:**

We revealed a positive relationship in this population between the number of influenza patients and the number of telephone triages for fever.

## Introduction

Seasonal influenza is a pandemic occurring every year that sometimes causes elderly people and infants to die. If seasonal influenza spreads, it not only harms people’s health but also causes social and economic damage due to absence from work. Replacing traditional surveillance with syndromic surveillance is one of the major interests in public health. Several syndromic surveillance models with absenteeism records [1–3], drug sales including over-the-counter drugs [4,5], and visits to the emergency department [6,7] have been reported previously. There is also a model to predict the influenza epidemic from search engine data on the Internet [3,8–11]. In Japan, according the Infectious Disease Control Law, patients diagnosed as having influenza are reported by the medical institution to the local health center, and the Department of Public Health of each local government officially announces the aggregate results of influenza. However, because this traditional surveillance takes time, a timelier surveillance system is needed to prevent an epidemic of influenza.

In Osaka prefecture, telephone triage service has been provided to the residents since 2012. The triage nurse uses software to determine the urgency of the client for each symptom and provides necessary services such as ambulance dispatch and guidance of medical institutions based on the result. Therefore, the number of telephone triages by symptom can be calculated in real time with this software.

If there is a relationship between the number of telephone triages for fever and the number of influenza patients, it may be possible for the software to predict an epidemic of influenza based on the number of telephone triages. The aim of this study was to clarify the relationship between the number of telephone triages for fever and the number of influenza patients in Osaka, Japan.

## Methods

### Study design, population and setting

This study was a retrospective, observational study conducted over a study period of six years from January 2012 to December 2017. Osaka prefecture is the largest urban area in western Japan, with an area of 1905.14 km^2^ and 2.3 million elderly people over 65 years old among the population of 8.8 million people [12]. In this study, we included the cases for which people called for telephone triage service and triage nurses judged the urgency of the client with software, and we excluded the cases for which nurses gave people information on accessible hospitals without doing telephone triage and cases not requiring health consultation. This study was approved by the ethics committee of Osaka University Graduate School of Medicine (approval no.: 16070). The requirement for informed consent was waved because the telephone triage data were anonymized. This article was written based on the STROBE statement to assess the reporting of cohort and cross-sectional studies [13].

### Outpatient surveillance of influenza-like illness in Japan

The surveillance program for infectious diseases in Japan, which was begun in 1981 and forms the basis for influenza surveillance of outpatients [14,15], was revised and updated to its current format following the revision of the Infectious Disease Control Law in 2014 [14–17]. The system requires mandatory reporting of nationally notifiable diseases and sentinel surveillance systems for various types of infectious diseases [18].

Influenza is included in the sentinel surveillance system. The numbers of influenza patients from 5000 medical institutions across Japan are reported weekly to local health centers. Sentinel sites were chosen on the basis of their geographic distribution, whether a clinic or hospital, and population densities. These sentinel sites report influenza-like illness according to the following criteria: (1) sudden onset of illness, (2) fever >38°C, (3) symptoms of upper respiratory inflammation, and (4) systemic symptoms such as general fatigue. A case meets the reporting criteria if the patient meets all four of the above symptoms or has at least one of the four symptoms along with a positive rapid diagnostic test [16]. Information on the age group and sex of the patients is reported every week by the sentinel sites and is then transferred from the local health centers to each prefectural government’s Department of Public Health, which aggregates it into a prefectural report. The report is then received by the National Institute of Infectious Diseases in Tokyo, which is affiliated with the Ministry of Health, Labour and Welfare. Within Osaka, 300 medical institutions report influenza patients to 10 local health centers [19]. In this study, the main endpoint was the weekly number of influenza patients in Osaka. These data were acquired from the website listed in [19].

### Telephone triage service in Osaka, Japan

As with the telephone triage service in Tokyo, that in Osaka prefecture is also a public service [20] and can be freely used by anyone. A triage nurse at the service uses software with a protocol for telephone triage in Japan and determines the urgency of the client. There are 97 different protocols of telephone triage for chief complaints in Japan, and the urgency of the client is determined by selecting the signs and symptoms related to each chief complaint. As with telephone triage service in the departments of veterans’ affairs in the United States [21], Canada and United Kingdom [22–24], the telephone service in Osaka provides the client necessary services such as ambulance dispatch and guidance of medical institutions based on the result of the urgency [25]. The software records the sex and age group of the client, the time when the telephone triage was started and ended, the chief complaint and selected signs and symptoms, the urgency of the client and whether an ambulance was dispatched.

### Statistical analysis

Using a linear regression model, we calculated the R^2^ of the regression model to assess the relationship between the number of telephone triages and the number of influenza patients in Osaka. The covariates in the linear regression model were the weekly number of telephone triages for fever and the week number. We defined the week number as a category variable having 52 values, with the week including January 1st as “week number = 1”. Next, to verify the validity of the regression model, we conducted a 6-fold cross-validation method based on 6 datasets decomposed by the calendar year. We evaluated it by the mean of Spearman’s correlation coefficient between the number of influenza patients and the predicted number of influenza patients in each test dataset. Lastly, according to the influenza outbreak season from December to April in Japan, we calculated the Spearman’s correlation coefficient and R^2^ between the predicted weekly number of influenza patients from the linear regression model and that of influenza patients for this season. Statistical significance was defined as *P*<0.05, and statistical analysis was performed by SPSS version 23.0J (IBM Crop., Armonk, NY).

## Results

Among a total of 1,065,628 telephone triages performed during the study period, the number of telephone triages for fever was 101,572. In addition, 465,971 influenza patients were reported from sentinel sites to the Department of Public Health in Osaka during the study period. Table 1 shows the characteristics of telephone triage in Osaka from 2012 to 2017. The age group with the highest number of telephone triages was that of 0–9 years old, for which the number was 542,890 (50.9%). The number of telephone triages was 511,267 (48.0%) for males and 553,000 (51.9%) for females. The greatest number of people who called for telephone triage were from the patient’s family, comprising 791,302 (74.3%) people. The number of telephone triages was 103,471 in 2012, and it had increased to 224,461 triages in 2017. The most common chief complaint during telephone triage was abnormal vital signs such as “no response” and “no breathing”, which comprised 407,754 (38.2%) triages. The number of telephone triages for fever was 101,572 (9.5%).

**Table 1:** Demographic and clinical characteristics of telephone triage between 2012 and 2017 in Osaka.

	Total	
Characteristic	(*n* = 1,065,628)	
Age group (years), n (%)		
0–9	542,890	(50.9)
10–19	59,327	(5.6)
20–29	69,479	(6.5)
30–39	85,391	(8.0)
40–49	75,383	(7.1)
50–59	45,547	(4.3)
60–69	58,482	(5.5)
70–79	69,128	(6.5)
80–89	46,518	(4.4)
90–99	10,630	(1.0)
100~	246	(0.0)
Unknown	2,597	(0.2)
Sex, n (%)		
Male	511,267	(48.0)
Female	553,000	(51.9)
Unknown	961	(0.1)
Person initiating the telephone consultation, n (%)		
Patient	252,693	(23.7)
Patient’s family	791,302	(74.3)
Other person	21,244	(2.0)
Unknown	389	(0.0)
Time of telephone consultation and triage, n (%)		
Daytime (9:00 to 17:59)	401,433	(37.7)
Nighttime (18:00 to 8:59)	664,195	(62.3)
Year, n (%)		
2012	103,471	(9.7)
2013	108,852	(10.2)
2014	196,972	(18.5)
2015	213,878	(20.1)
2016	217,994	(20.5)
2017	224,461	(21.1)
Season, n (%)		
Winter (January to March)	260,818	(24.5)
Spring (April to June)	263,609	(24.7)
Summer (July to September)	270,805	(25.4)
Autumn (October to December)	270,396	(25.4)
Contents of telephone consultation and triage for chief complaint, n (%)		
Abnormal vital signs	407,754	(38.2)
Fever	101,572	(9.5)
Nausea, vomiting	54,283	(5.1)
Head injury	50,508	(4.7)
Stomachache	33,005	(3.1)
Rash/Hives	32,644	(3.1)
Headache	23,172	(2.2)
Vertigo/Flutter	20,426	(1.9)
Accidental ingestion of solids	18,179	(1.7)
Numbness/Paralysis	13,082	(1.2)
Chest pain	12,834	(1.2)
Other	298,169	(28.0)

**Fig 1:**
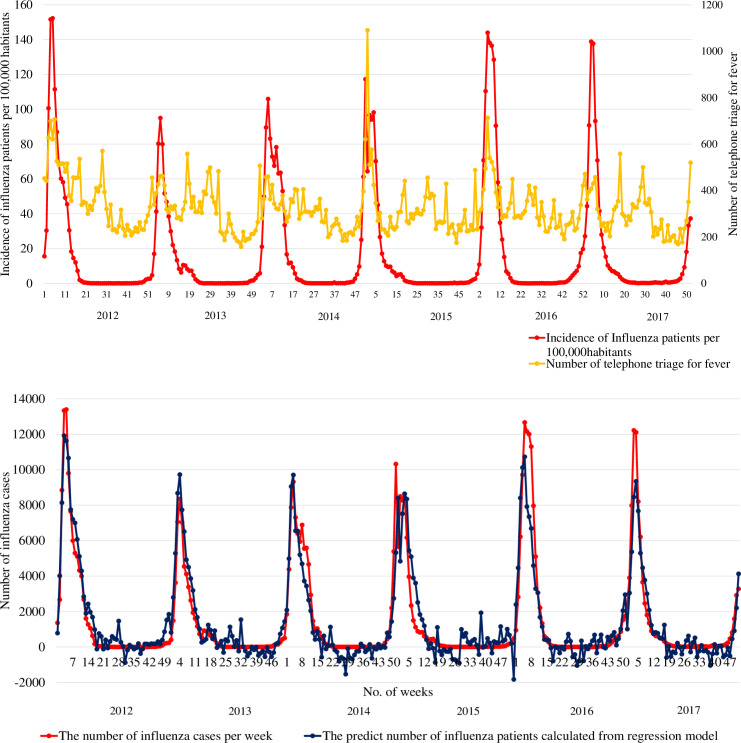
Weekly and predicted numbers of influenza patients and telephone triages. **a.** The weekly number of influenza patients and the weekly number of telephone triages for fever during the study period. **b.** The weekly number of influenza patients and the predicted number of influenza patients from the linear regression model during the study period.

Fig 1A shows the relationship between the weekly incidence of influenza patients and the weekly number of telephone triages for fever. Fig 1B shows the weekly number of influenza patients and the predicted number of influenza patients from the linear regression model. The red line indicates the weekly number of influenza patients, the yellow line the weekly number of telephone triages for fever and the blue line the predicted number of influenza patients from the linear regression model. The R^2^ of this linear regression model was 0.869, and the adjusted R^2^ was 0.842.

**Fig 2:**
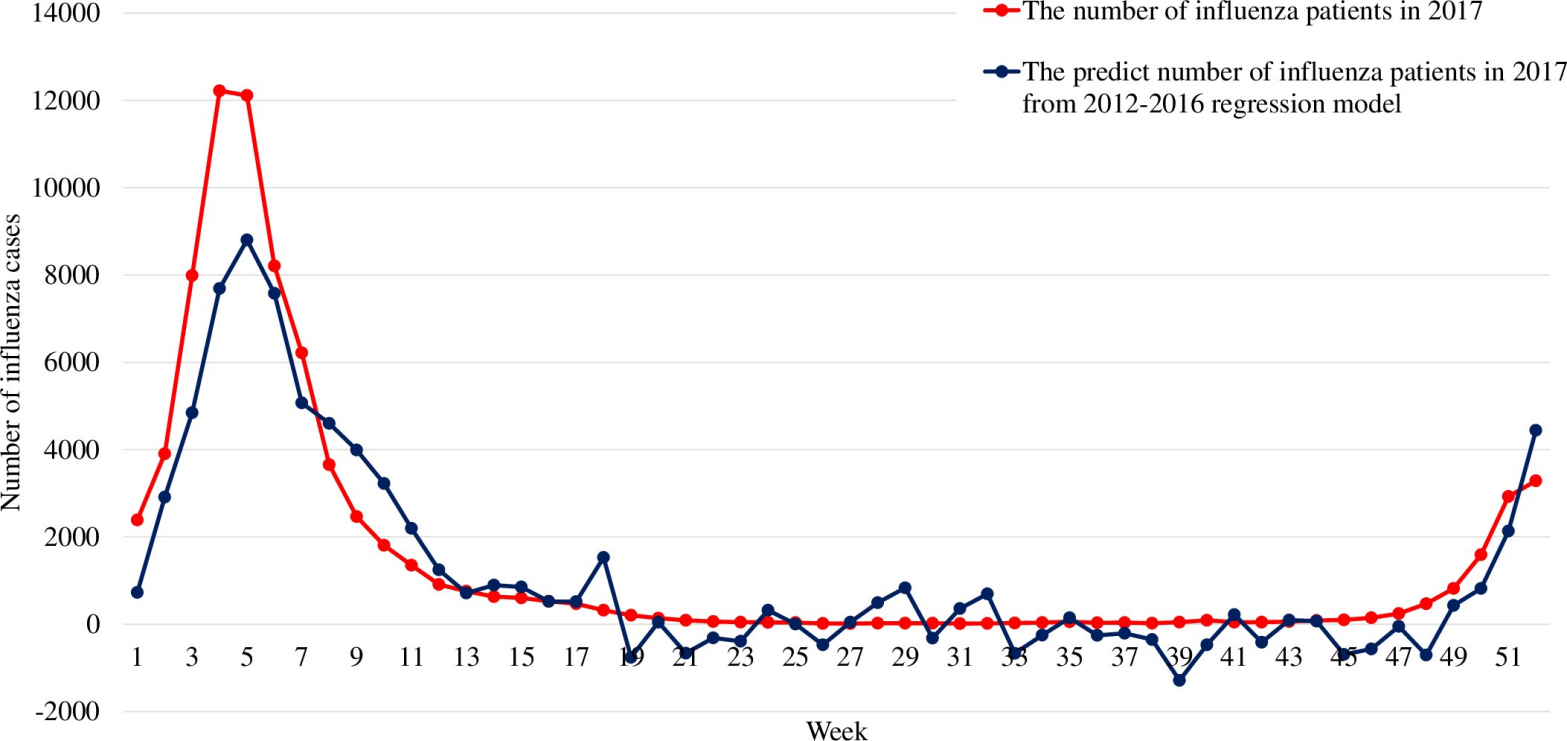
The weekly number of influenza patients in 2017 and the predicted number of influenza patients in 2017 from the linear regression model from 2012–2016.

Fig 2 shows the weekly number of influenza patients in 2017 and the predicted number of influenza patients from the 2012–2016 linear regression model. The R^2^ of this 2012–2016 linear regression model was 0.869, and the adjusted R^2^ was 0.835. The mean of Spearman’s correlation coefficient between the number of influenza patients and the predicted number of influenza patients from this linear regression model was 0.724.

**Fig 3:**
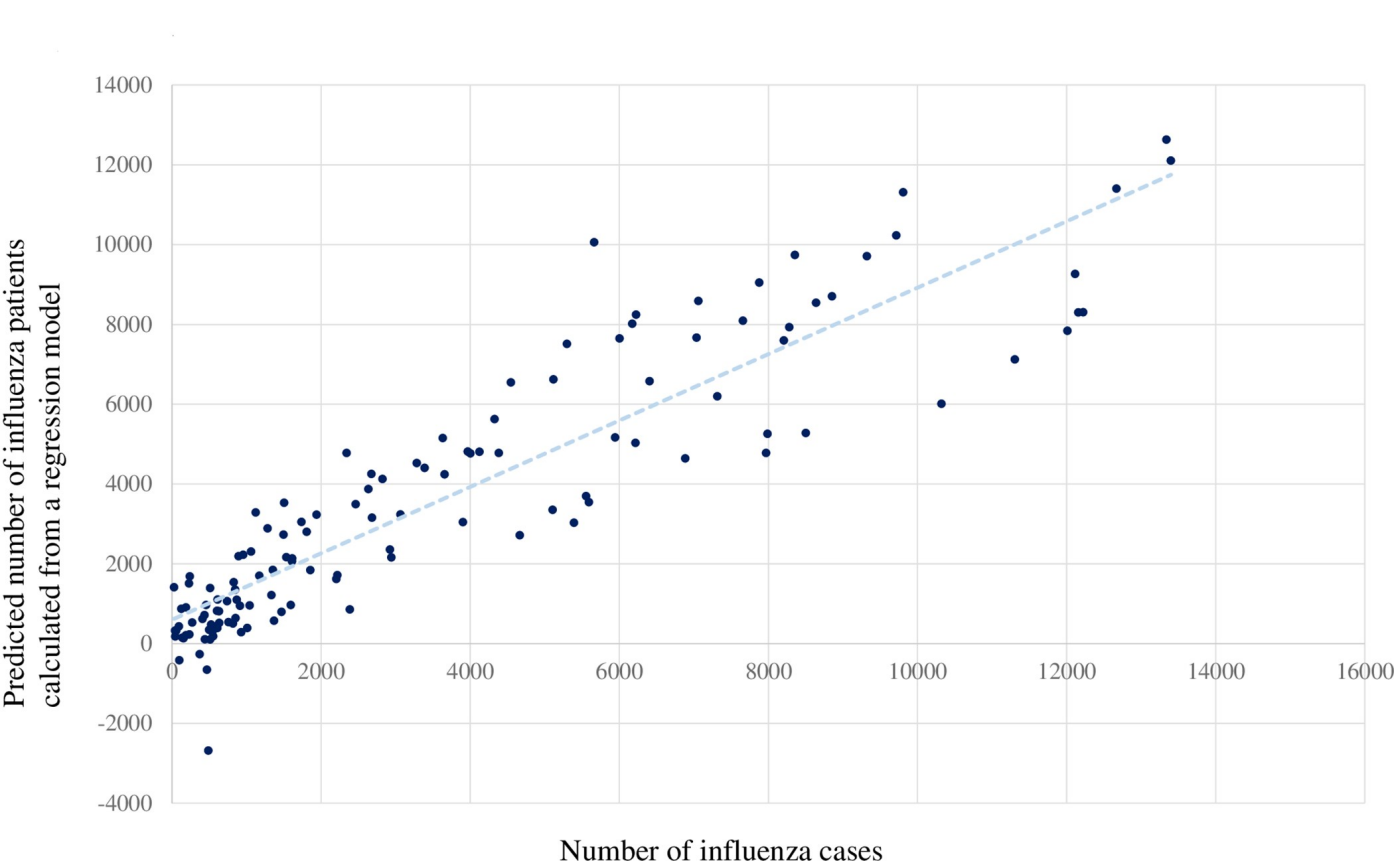
Scatter plot of the predicted number of influenza patients from the linear regression model and the weekly number of influenza patients during the influenza season from December to April.

Fig 3 shows a scatter plot of the predicted number of influenza patients in the influenza outbreak season from the linear regression model and the weekly number of influenza patients from December to April. Spearman’s correlation coefficient was 0.923 (P<0.001).

## Discussion

We revealed a positive relationship between the number of telephone triages for fever and the number of influenza patients in a large metropolitan area of Japan in this study. We also found a difference in the contribution rate of the linear regression model depending on the target period. Furthermore, the predicted number of influenza patients from the linear regression model correlated well with the weekly number of influenza patients in the influenza season from December to April in Japan. The contribution rate of this linear regression model using telephone triage data was very high. The prediction of an influenza epidemic using this linear regression model could potentially allow an official announcement to be made earlier than with the previous aggregate calculation from the public service, and this may lead to the prevention of a pandemic of influenza.

A number of studies have predicted the number of patients with influenza-like disease and respiratory disease using telephone triage data and the number of patients visiting the emergency room [3,6,21–23,26–29]. Perry and colleagues compared the prediction performance of the number of patients with respiratory disease visiting the emergency room using N4SID (numerical methods for subspace state space identification), EWMA (exponentially weighted moving average), FOS (fast orthogonal search), and a regression model [7]. As a result, they revealed that the FOS model had better prediction accuracy than the regression model if the population was large, but the regression model had the highest prediction accuracy in areas with less population. Lucero-Obusan et al. also reported in a study of the Department of Veterans’ Affairs in the United States that telephone triage data and influenza-related indicators were significantly correlated [21]. In the present study, we used a linear regression model in which the number of telephone triages for fever and seasonality as defined by the week number were input as covariates. Influenza is a seasonal disease that mainly spreads from autumn to winter, and fever is one of the main symptoms of influenza. If influenza is prevalent among people, the number of telephone triages for fever increases at a constant rate, which leads to the high contribution rate of this regression model. In addition, this study was conducted in an area with 8.8 million people over six years, and the validity of this regression model was also very high.

In this study, by using a 6-fold cross-validation method with calendar year, we found a high correlation between the number of influenza patients and the predicted number of influenza patients from a linear regression model. Although there were some differences in the correlation coefficients in each year, the coefficients were generally high enough to predict an epidemic of influenza. However, it is unclear whether prediction from this predictive model could transform public behavior and control the spread of influenza. Further research would be needed.

## Limitations

There are some limitations in this study. First, the report of influenza is a fixed-point observation based on the Infectious Disease Control Law, and influenza surveillance is not a survey of all cases. Second, the criteria for reporting influenza patients in Japan includes patients diagnosed as having influenza by clinical symptoms [30]. In Japan, the diagnosis of influenza is mostly performed using a diagnostic kit. However, as some patients with influenza were diagnosed by their clinical symptoms, there might be a small number of non-influenza patients among the reported number of influenza patients. Third, as about half of the subjects of this study were children, there might be selection bias compared to the actual population structure of Japan [12]. Fourth, we could not perform a geographic analysis because the telephone triage data did not include geographic address data. Fifth, although information on the age groups and sex of the patients has been reported every week by the sentinel sites, the number of patients with infectious diseases by sex has not been disclosed in Japan. Therefore, we could not conduct a sub-group analysis separated by sex. Finally, this study was an observational study, and there may be some unknown confounding factors.

## Conclusions

In this study, we revealed a positive relationship between the number of influenza patients and the number of telephone triages for fever in a large metropolitan area in Japan. Especially, the actual number of influenza patients and the predicted number of influenza cases using this regression model correlated well in the influenza season from December to April.

## Supporting information

S1 File (ZIP)
