# Supplementary material for: The relationship between seasonal influenza and telephone triage for fever: A population-based study in Osaka, Japan
Source: PLoS One. 2020 Aug 6;15(8):e0236560. doi: 10.1371/journal.pone.0236560 (PMC7410252; doi:10.1371/journal.pone.0236560)
Supplement: S1 File — (ZIP) [file pone.0236560.s001.zip › Age group/Figure 4_0-4 years old.pptx]

## Slide 1
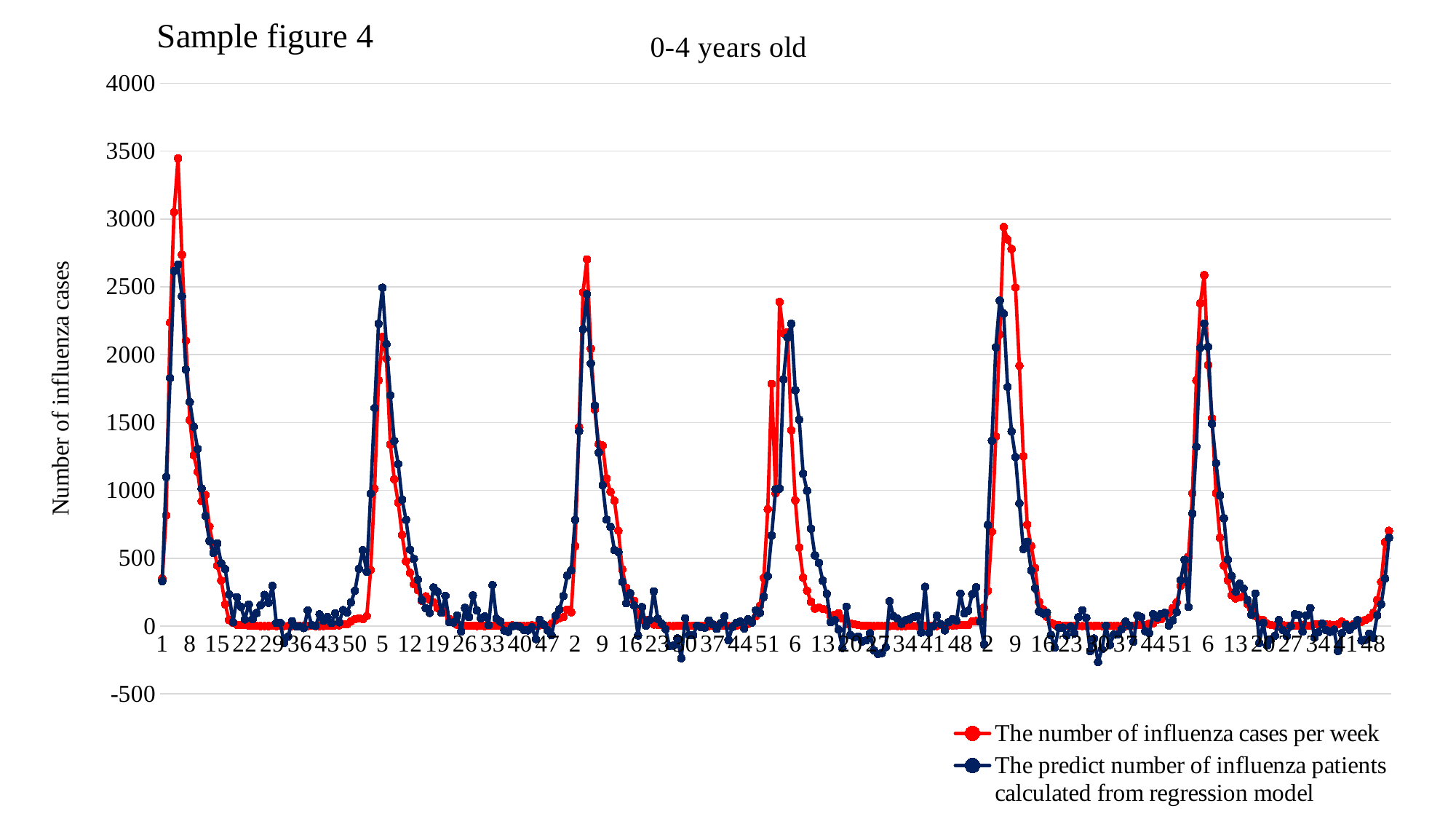

### Chart: 0-4 years old
| Category | The number of influenza cases per week | The predict number of influenza patients calculated from regression model |
|---|---|---|
| 1 | 352.0 | 331.2806007963919 |
| 2 | 817.0 | 1098.2726851956672 |
| 3 | 2237.0 | 1828.3199900639788 |
| 4 | 3051.0 | 2615.413766629576 |
| 5 | 3447.0 | 2663.8251373679373 |
| 6 | 2736.0 | 2431.734618005235 |
| 7 | 2102.0 | 1891.3974084677598 |
| 8 | 1517.0 | 1652.469604757523 |
| 9 | 1260.0 | 1469.5668179315335 |
| 10 | 1136.0 | 1305.0101353763953 |
| 11 | 921.0 | 1013.1629483512498 |
| 12 | 967.0 | 811.624741801099 |
| 13 | 734.0 | 626.8383293500038 |
| 14 | 589.0 | 539.9015752967349 |
| 15 | 445.0 | 610.2541237956285 |
| 16 | 336.0 | 462.3894660689738 |
| 17 | 160.0 | 419.65430950039615 |
| 18 | 44.0 | 232.7356627053033 |
| 19 | 24.0 | 27.509000000000015 |
| 20 | 9.0 | 213.21514762833942 |
| 21 | 8.0 | 141.43255577173102 |
| 22 | 7.0 | 48.6896226375527 |
| 23 | 2.0 | 159.386672619126 |
| 24 | 1.0 | 50.585918565857014 |
| 25 | 1.0 | 96.00688447275695 |
| 26 | 0.0 | 154.22490930124184 |
| 27 | 0.0 | 229.90217576875364 |
| 28 | 0.0 | 171.32145912051385 |
| 29 | 1.0 | 295.9543614735204 |
| 30 | 0.0 | 23.161188345297433 |
| 31 | 1.0 | 23.521164837190668 |
| 32 | 0.0 | -125.302235361964 |
| 33 | 1.0 | -78.71202350833357 |
| 34 | 0.0 | 35.54347135048897 |
| 35 | 0.0 | -1.8104561556064027 |
| 36 | 1.0 | -2.6425650587649443 |
| 37 | 1.0 | -15.27091659672476 |
| 38 | 0.0 | 115.36158886905571 |
| 39 | 2.0 | 6.756174101806209 |
| 40 | 0.0 | 0.9544951831987873 |
| 41 | 0.0 | 86.66468552027379 |
| 42 | 0.0 | 39.80843394546025 |
| 43 | 2.0 | 67.50110292828106 |
| 44 | 2.0 | 22.54832967460959 |
| 45 | 6.0 | 94.16467426410884 |
| 46 | 5.0 | 27.58393510353733 |
| 47 | 12.0 | 118.06409366366609 |
| 48 | 12.0 | 99.4071492733218 |
| 49 | 36.0 | 174.36288743811804 |
| 50 | 51.0 | 258.89432040999407 |
| 51 | 57.0 | 422.0633055172956 |
| 52 | 51.0 | 558.8135014824526 |
| 1 | 74.0 | 399.26060079639194 |
| 2 | 414.0 | 975.9086851956671 |
| 3 | 1012.0 | 1607.3849900639789 |
| 4 | 1811.0 | 2227.927766629576 |
| 5 | 2131.0 | 2493.8751373679374 |
| 6 | 1971.0 | 2078.238618005235 |
| 7 | 1338.0 | 1701.0534084677597 |
| 8 | 1082.0 | 1363.554604757523 |
| 9 | 910.0 | 1194.2478179315335 |
| 10 | 671.0 | 931.1201353763955 |
| 11 | 477.0 | 782.0309483512498 |
| 12 | 392.0 | 563.4977418010991 |
| 13 | 308.0 | 494.2773293500037 |
| 14 | 266.0 | 342.75957529673485 |
| 15 | 186.0 | 192.1771237956283 |
| 16 | 219.0 | 132.6864660689739 |
| 17 | 193.0 | 96.7493095003963 |
| 18 | 174.0 | 283.7206627053032 |
| 19 | 133.0 | 251.84299999999996 |
| 20 | 131.0 | 97.64914762833928 |
| 21 | 101.0 | 223.00855577173104 |
| 22 | 52.0 | 28.295622637552697 |
| 23 | 35.0 | 26.82567261912584 |
| 24 | 10.0 | 77.77791856585702 |
| 25 | 5.0 | -39.953115527243085 |
| 26 | 2.0 | 133.83090930124195 |
| 27 | 2.0 | 66.7501757687537 |
| 28 | 2.0 | 225.70545912051386 |
| 29 | 0.0 | 115.80736147352047 |
| 30 | 4.0 | 57.15118834529744 |
| 31 | 0.0 | 71.10716483719068 |
| 32 | 2.0 | 7.258764638035927 |
| 33 | 1.0 | 301.9759764916663 |
| 34 | 2.0 | 55.93747135048898 |
| 35 | 1.0 | 32.179543844393606 |
| 36 | 2.0 | -33.23356505876484 |
| 37 | 4.0 | -42.46291659672477 |
| 38 | 6.0 | -3.6034111309443233 |
| 39 | 1.0 | 3.3571741018062085 |
| 40 | 1.0 | -5.8435048168012145 |
| 41 | 1.0 | -28.90131447972624 |
| 42 | 2.0 | -31.57056605453988 |
| 43 | 6.0 | -7.276897071718963 |
| 44 | 2.0 | -96.41667032539044 |
| 45 | 6.0 | 46.57867426410883 |
| 46 | 7.0 | 10.588935103537437 |
| 47 | 4.0 | -31.491906336333955 |
| 48 | 19.0 | -67.14385072667812 |
| 49 | 43.0 | 75.7918874381179 |
| 50 | 59.0 | 122.93432040999414 |
| 51 | 66.0 | 221.52230551729565 |
| 52 | 122.0 | 371.86850148245264 |
| 1 | 102.0 | 409.45760079639183 |
| 2 | 588.0 | 782.1656851956672 |
| 3 | 1466.0 | 1437.4349900639786 |
| 4 | 2458.0 | 2187.139766629576 |
| 5 | 2703.0 | 2446.289137367937 |
| 6 | 2045.0 | 1935.4806180052346 |
| 7 | 1594.0 | 1626.27540846776 |
| 8 | 1339.0 | 1278.5796047575232 |
| 9 | 1330.0 | 1037.8938179315337 |
| 10 | 1087.0 | 784.9631353763958 |
| 11 | 989.0 | 731.0459483512499 |
| 12 | 924.0 | 560.0987418010992 |
| 13 | 702.0 | 545.2623293500038 |
| 14 | 418.0 | 325.76457529673473 |
| 15 | 282.0 | 168.3841237956284 |
| 16 | 240.0 | 241.45446606897383 |
| 17 | 186.0 | 151.1333095003962 |
| 18 | 86.0 | -69.77533729469667 |
| 19 | 48.0 | 139.67600000000004 |
| 20 | 44.0 | 2.477147628339253 |
| 21 | 30.0 | 46.26055577173099 |
| 22 | 11.0 | 256.02862263755276 |
| 23 | 8.0 | 54.01767261912585 |
| 24 | 7.0 | 16.595918565857005 |
| 25 | 0.0 | -22.95811552724308 |
| 26 | 1.0 | -148.28609069875802 |
| 27 | 0.0 | -143.98782423124635 |
| 28 | 3.0 | -90.40154087948622 |
| 29 | 1.0 | -237.6886385264795 |
| 30 | 1.0 | 57.15118834529744 |
| 31 | 1.0 | -68.25183516280924 |
| 32 | 1.0 | -67.51923536196398 |
| 33 | 1.0 | 2.863976491666449 |
| 34 | 2.0 | -8.64352864951104 |
| 35 | 3.0 | -12.007456155606405 |
| 36 | 0.0 | 41.54443494123518 |
| 37 | 2.0 | 11.921083403275247 |
| 38 | 1.0 | -20.598411130944328 |
| 39 | 2.0 | 20.352174101806213 |
| 40 | 1.0 | 72.3334951831988 |
| 41 | 1.0 | -103.67931447972626 |
| 42 | 0.0 | -7.777566054539761 |
| 43 | 2.0 | 23.31410292828093 |
| 44 | 9.0 | 32.74532967460959 |
| 45 | 12.0 | -18.002325735891304 |
| 46 | 16.0 | 51.37693510353745 |
| 47 | 36.0 | 26.29109366366606 |
| 48 | 72.0 | 116.40214927332192 |
| 49 | 150.0 | 96.1858874381179 |
| 50 | 355.0 | 214.70732040999417 |
| 51 | 861.0 | 367.6793055172956 |
| 52 | 1785.0 | 667.5815014824526 |
| 1 | 978.0 | 1007.6816007963918 |
| 2 | 2389.0 | 1013.297685195667 |
| 3 | 2157.0 | 1818.1229900639787 |
| 4 | 2166.0 | 2125.9577666295763 |
| 5 | 1442.0 | 2228.753137367937 |
| 6 | 927.0 | 1738.3386180052348 |
| 7 | 579.0 | 1520.9064084677598 |
| 8 | 357.0 | 1122.225604757523 |
| 9 | 261.0 | 997.1058179315338 |
| 10 | 179.0 | 716.9831353763958 |
| 11 | 128.0 | 520.3079483512498 |
| 12 | 136.0 | 464.9267418010992 |
| 13 | 127.0 | 334.524329350004 |
| 14 | 123.0 | 237.39057529673494 |
| 15 | 76.0 | 29.025123795628474 |
| 16 | 84.0 | 44.31246606897389 |
| 17 | 94.0 | -25.61469049960374 |
| 18 | 55.0 | -161.54833729469658 |
| 19 | 16.0 | 143.07500000000005 |
| 20 | 20.0 | -65.50285237166077 |
| 21 | 12.0 | -82.90144422826904 |
| 22 | 7.0 | -77.07337736244733 |
| 23 | 2.0 | -112.5333273808742 |
| 24 | 2.0 | -105.76808143414303 |
| 25 | 1.0 | -53.54911552724309 |
| 26 | 0.0 | -178.87709069875802 |
| 27 | 2.0 | -205.16982423124637 |
| 28 | 1.0 | -199.16954087948625 |
| 29 | 2.0 | -156.1126385264795 |
| 30 | 0.0 | 182.91418834529725 |
| 31 | 1.0 | 71.10716483719068 |
| 32 | 0.0 | 54.84476463803594 |
| 33 | 0.0 | 16.459976491666453 |
| 34 | 0.0 | 42.34147135048897 |
| 35 | 2.0 | 49.17454384439361 |
| 36 | 1.0 | 65.33743494123507 |
| 37 | 0.0 | 73.10308340327526 |
| 38 | 1.0 | -47.79041113094422 |
| 39 | 0.0 | 288.87317410180606 |
| 40 | 1.0 | -50.030504816801226 |
| 41 | 1.0 | -1.7093144797262312 |
| 42 | 0.0 | 77.19743394546015 |
| 43 | 7.0 | 13.117102928281042 |
| 44 | 3.0 | -31.835670325390424 |
| 45 | 4.0 | 29.58367426410871 |
| 46 | 4.0 | 51.37693510353745 |
| 47 | 6.0 | 39.887093663666064 |
| 48 | 9.0 | 238.76614927332173 |
| 49 | 7.0 | 92.7868874381179 |
| 50 | 8.0 | 112.73732040999414 |
| 51 | 34.0 | 235.11830551729565 |
| 52 | 37.0 | 286.8935014824525 |
| 53 | 33.0 | 33.03771764379451 |
| 1 | 137.0 | -134.3823992036082 |
| 2 | 259.0 | 744.7766851956673 |
| 3 | 696.0 | 1366.0559900639787 |
| 4 | 1397.0 | 2054.578766629576 |
| 5 | 2150.0 | 2398.703137367937 |
| 6 | 2941.0 | 2302.5726180052347 |
| 7 | 2849.0 | 1762.23540846776 |
| 8 | 2779.0 | 1434.933604757523 |
| 9 | 2495.0 | 1245.2328179315336 |
| 10 | 1919.0 | 903.9281353763955 |
| 11 | 1252.0 | 567.8939483512501 |
| 12 | 746.0 | 621.280741801099 |
| 13 | 589.0 | 409.3023293500038 |
| 14 | 428.0 | 278.17857529673495 |
| 15 | 177.0 | 107.20212379562838 |
| 16 | 123.0 | 91.8984660689739 |
| 17 | 67.0 | 100.1483095003963 |
| 18 | 24.0 | -66.37633729469678 |
| 19 | 10.0 | -159.43600000000004 |
| 20 | 9.0 | -14.517852371660751 |
| 21 | 4.0 | -11.522444228269137 |
| 22 | 3.0 | -70.27537736244733 |
| 23 | 0.0 | -3.7653273808741687 |
| 24 | 1.0 | -54.78308143414313 |
| 25 | 1.0 | 65.41588447275694 |
| 26 | 0.0 | 116.83590930124205 |
| 27 | 0.0 | 59.952175768753705 |
| 28 | 1.0 | -185.57354087948625 |
| 29 | 0.0 | -91.53163852647947 |
| 30 | 2.0 | -265.75381165470253 |
| 31 | 0.0 | -170.22183516280927 |
| 32 | 0.0 | 3.8597646380359265 |
| 33 | 1.0 | -139.89402350833348 |
| 34 | 0.0 | -63.027528649511055 |
| 35 | 0.0 | -62.99245615560642 |
| 36 | 1.0 | -23.03656505876495 |
| 37 | 0.0 | 32.31508340327525 |
| 38 | 4.0 | 3.1945888690556785 |
| 39 | 3.0 | -115.60782589819382 |
| 40 | 8.0 | 79.13149518319881 |
| 41 | 10.0 | 66.27068552027379 |
| 42 | 8.0 | -38.36856605453988 |
| 43 | 18.0 | -51.46389707171909 |
| 44 | 21.0 | 87.12932967460961 |
| 45 | 46.0 | 60.17467426410883 |
| 46 | 51.0 | 88.76593510353734 |
| 47 | 75.0 | 97.67009366366608 |
| 48 | 95.0 | 4.235149273321781 |
| 49 | 133.0 | 41.80188743811789 |
| 50 | 174.0 | 102.54032040999414 |
| 51 | 297.0 | 337.08830551729545 |
| 52 | 326.0 | 487.4345014824527 |
| 1 | 511.0 | 140.93660079639176 |
| 2 | 977.0 | 829.7516851956672 |
| 3 | 1811.0 | 1321.8689900639788 |
| 4 | 2379.0 | 2051.179766629576 |
| 5 | 2587.0 | 2228.753137367937 |
| 6 | 1924.0 | 2057.8446180052347 |
| 7 | 1530.0 | 1490.3154084677599 |
| 8 | 978.0 | 1200.402604757523 |
| 9 | 651.0 | 963.1158179315336 |
| 10 | 445.0 | 795.1601353763957 |
| 11 | 337.0 | 489.7169483512499 |
| 12 | 226.0 | 369.75474180109916 |
| 13 | 203.0 | 252.94832935000397 |
| 14 | 212.0 | 312.16857529673473 |
| 15 | 218.0 | 277.1521237956284 |
| 16 | 161.0 | 190.46946606897393 |
| 17 | 125.0 | 83.15330950039629 |
| 18 | 75.0 | 239.5336627053033 |
| 19 | 46.0 | -125.44600000000003 |
| 20 | 43.0 | 22.87114762833926 |
| 21 | 17.0 | -144.08344422826906 |
| 22 | 8.0 | -97.46737736244734 |
| 23 | 5.0 | -71.74532738087419 |
| 24 | 7.0 | 43.78791856585701 |
| 25 | 7.0 | -29.756115527243082 |
| 26 | 1.0 | -73.508090698758 |
| 27 | 2.0 | -1.2298242312463117 |
| 28 | 1.0 | 86.34645912051371 |
| 29 | 4.0 | 81.81736147352046 |
| 30 | 6.0 | -41.419811654702585 |
| 31 | 2.0 | 77.90516483719068 |
| 32 | 3.0 | 133.02176463803596 |
| 33 | 13.0 | -85.51002350833357 |
| 34 | 12.0 | -46.03252864951105 |
| 35 | 17.0 | 18.583543844393603 |
| 36 | 13.0 | -29.83456505876495 |
| 37 | 10.0 | -42.46291659672477 |
| 38 | 7.0 | -27.39641113094433 |
| 39 | 12.0 | -183.58782589819384 |
| 40 | 32.0 | -53.42950481680123 |
| 41 | 14.0 | 8.487685520273772 |
| 42 | 1.0 | -28.171566054539767 |
| 43 | 13.0 | 2.920102928280926 |
| 44 | 20.0 | 42.942329674609596 |
| 45 | 32.0 | -106.37632573589133 |
| 46 | 45.0 | -101.5780648964627 |
| 47 | 62.0 | -55.28490633633396 |
| 48 | 97.0 | -87.53785072667813 |
| 49 | 191.0 | 79.1908874381179 |
| 50 | 325.0 | 160.32332040999415 |
| 51 | 619.0 | 350.68430551729546 |
| 52 | 702.0 | 650.5865014824527 |
