# Supplementary material for: The relationship between seasonal influenza and telephone triage for fever: A population-based study in Osaka, Japan
Source: PLoS One. 2020 Aug 6;15(8):e0236560. doi: 10.1371/journal.pone.0236560 (PMC7410252; doi:10.1371/journal.pone.0236560)
Supplement: S1 File — (ZIP) [file pone.0236560.s001.zip › Age group/Figure 4_10-14 years old.pptx]

## Slide 1
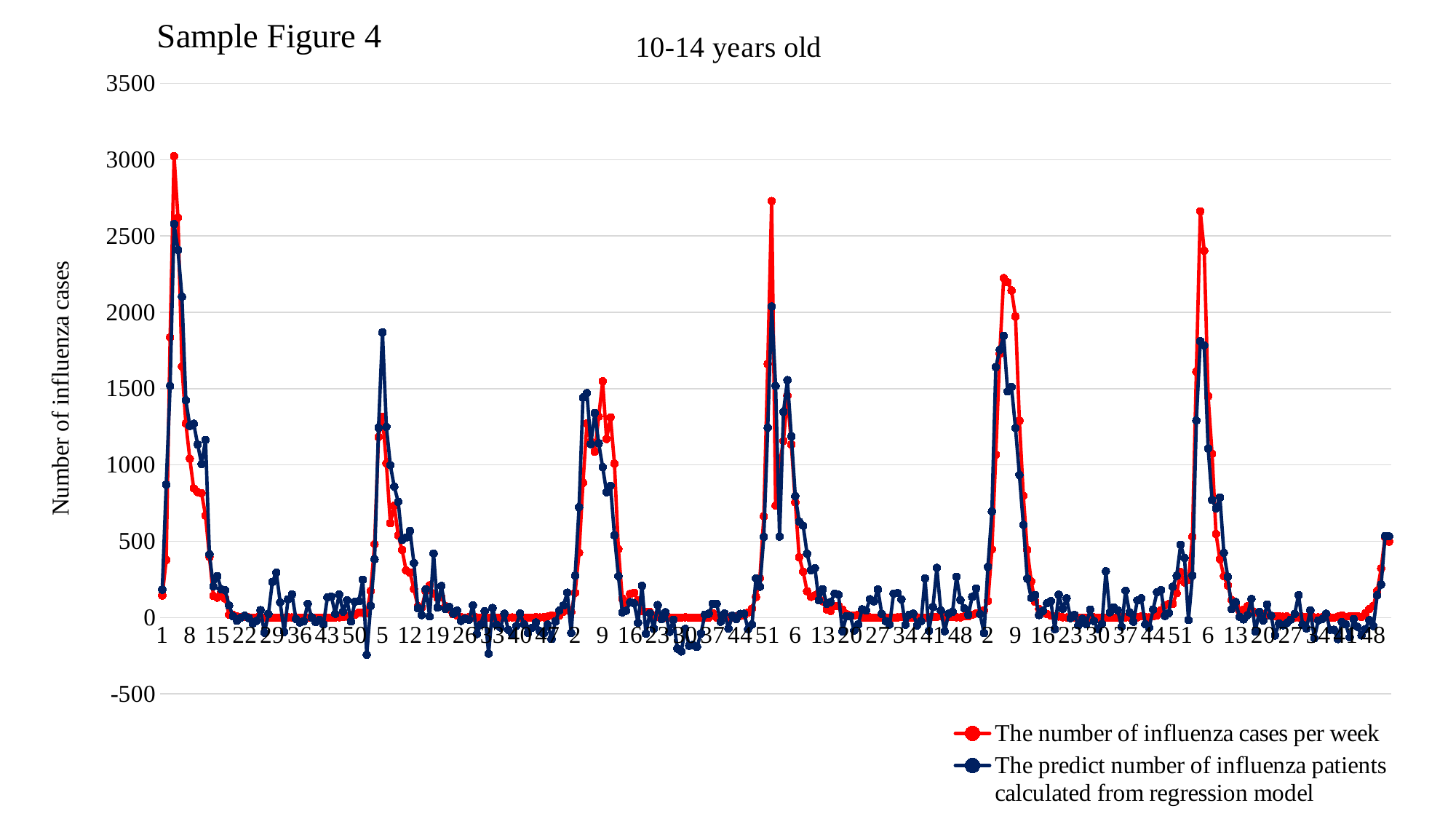

### Chart: 10-14 years old
| Category | The number of influenza cases per week | The predict number of influenza patients calculated from regression model |
|---|---|---|
| 1 | 145.0 | 182.49552140137877 |
| 2 | 377.0 | 871.6330396477813 |
| 3 | 1836.0 | 1518.4727024533572 |
| 4 | 3023.0 | 2578.320539294759 |
| 5 | 2620.0 | 2408.2585991195765 |
| 6 | 1645.0 | 2101.701244031768 |
| 7 | 1272.0 | 1424.5100901728863 |
| 8 | 1041.0 | 1255.2662968404363 |
| 9 | 846.0 | 1269.7830342091604 |
| 10 | 822.0 | 1133.6617335075455 |
| 11 | 815.0 | 1005.6231864905135 |
| 12 | 668.0 | 1163.3331484201758 |
| 13 | 397.0 | 413.75670473691906 |
| 14 | 141.0 | 203.9488352637929 |
| 15 | 131.0 | 272.1607700003564 |
| 16 | 167.0 | 186.00938771944527 |
| 17 | 124.0 | 178.34275368449715 |
| 18 | 19.0 | 79.20128982429026 |
| 19 | 17.0 | 9.02800000000002 |
| 20 | 7.0 | -20.14186403459169 |
| 21 | 4.0 | 0.16287333413112037 |
| 22 | 4.0 | 12.598862456891595 |
| 23 | 1.0 | -3.301842280112794 |
| 24 | 0.0 | -38.939815087014466 |
| 25 | 1.0 | -22.003825964253707 |
| 26 | 1.0 | 48.83945842150217 |
| 27 | 0.0 | -99.08121368378409 |
| 28 | 0.0 | 24.169365964966687 |
| 29 | 0.0 | 232.79857964866608 |
| 30 | 0.0 | 294.84058508728543 |
| 31 | 0.0 | 99.58002175439395 |
| 32 | 0.0 | -94.18067210537123 |
| 33 | 2.0 | 119.68257964866581 |
| 34 | 1.0 | 151.82081350931435 |
| 35 | 1.0 | -8.801809648394624 |
| 36 | 0.0 | -31.80513754310823 |
| 37 | 0.0 | -23.07122999964338 |
| 38 | 0.0 | 90.2787101755394 |
| 39 | 0.0 | 1.3327700003566179 |
| 40 | 0.0 | -29.638503508160056 |
| 41 | 0.0 | -15.769787893915748 |
| 42 | 0.0 | -44.00721912240388 |
| 43 | 0.0 | 132.31751824631925 |
| 44 | 0.0 | 138.05149105322056 |
| 45 | 1.0 | 25.10215771988726 |
| 46 | 2.0 | 151.7181577198872 |
| 47 | 4.0 | 39.56671017553958 |
| 48 | 7.0 | 115.04137684220615 |
| 49 | 19.0 | -25.017202806544844 |
| 50 | 17.0 | 102.81929526290986 |
| 51 | 32.0 | 107.5871647360355 |
| 52 | 31.0 | 248.0835431558557 |
| 1 | 29.0 | -243.56447859862126 |
| 2 | 175.0 | 76.32103964778116 |
| 3 | 481.0 | 382.3127024533572 |
| 4 | 1183.0 | 1243.3325392947588 |
| 5 | 1315.0 | 1868.5825991195768 |
| 6 | 1010.0 | 1249.5812440317684 |
| 7 | 619.0 | 998.4500901728863 |
| 8 | 733.0 | 857.6102968404364 |
| 9 | 539.0 | 758.5110342091602 |
| 10 | 443.0 | 508.7737335075455 |
| 11 | 310.0 | 522.7551864905136 |
| 12 | 295.0 | 566.8491484201757 |
| 13 | 187.0 | 356.94870473691907 |
| 14 | 74.0 | 61.928835263792905 |
| 15 | 67.0 | 16.524770000356398 |
| 16 | 172.0 | 186.00938771944527 |
| 17 | 211.0 | 7.918753684497119 |
| 18 | 157.0 | 420.04928982429027 |
| 19 | 130.0 | 65.83600000000001 |
| 20 | 133.0 | 207.0901359654083 |
| 21 | 76.0 | 56.97087333413111 |
| 22 | 54.0 | 69.40686245689159 |
| 23 | 22.0 | 25.102157719887202 |
| 24 | 12.0 | 46.27218491298555 |
| 25 | 2.0 | -22.003825964253707 |
| 26 | 0.0 | -7.96854157849782 |
| 27 | 2.0 | -13.869213683784096 |
| 28 | 1.0 | 80.97736596496668 |
| 29 | 1.0 | -108.04942035133394 |
| 30 | 0.0 | -46.00741491271452 |
| 31 | 0.0 | 42.77202175439396 |
| 32 | 0.0 | -236.2006721053712 |
| 33 | 0.0 | 62.874579648665815 |
| 34 | 0.0 | -47.007186490685655 |
| 35 | 0.0 | -65.60980964839462 |
| 36 | 1.0 | 25.002862456891762 |
| 37 | 0.0 | -79.87922999964337 |
| 38 | 1.0 | -108.5492898244606 |
| 39 | 0.0 | -55.475229999643375 |
| 40 | 0.0 | 27.169496491839936 |
| 41 | 0.0 | -44.173787893915744 |
| 42 | 0.0 | -100.8152191224039 |
| 43 | 0.0 | -66.51048175368072 |
| 44 | 3.0 | -32.37250894677942 |
| 45 | 1.0 | -88.51384228011275 |
| 46 | 2.0 | -103.91784228011281 |
| 47 | 6.0 | -45.645289824460434 |
| 48 | 13.0 | -140.59462315779385 |
| 49 | 11.0 | -25.017202806544844 |
| 50 | 14.0 | 46.01129526290987 |
| 51 | 39.0 | 79.18316473603551 |
| 52 | 48.0 | 162.87154315585565 |
| 1 | 37.0 | -101.54447859862128 |
| 2 | 162.0 | 275.1490396477812 |
| 3 | 426.0 | 723.1607024533573 |
| 4 | 883.0 | 1442.1605392947588 |
| 5 | 1272.0 | 1470.9265991195768 |
| 6 | 1152.0 | 1135.9652440317684 |
| 7 | 1087.0 | 1339.2980901728863 |
| 8 | 1317.0 | 1141.6502968404363 |
| 9 | 1548.0 | 985.7430342091604 |
| 10 | 1170.0 | 821.2177335075455 |
| 11 | 1312.0 | 863.6031864905135 |
| 12 | 1009.0 | 538.4451484201757 |
| 13 | 450.0 | 271.7367047369191 |
| 14 | 125.0 | 33.52483526379285 |
| 15 | 89.0 | 44.928770000356394 |
| 16 | 155.0 | 100.79738771944523 |
| 17 | 162.0 | 93.13075368449711 |
| 18 | 114.0 | -34.41471017570976 |
| 19 | 41.0 | 207.856 |
| 20 | 37.0 | -105.35386403459171 |
| 21 | 36.0 | 28.566873334131117 |
| 22 | 15.0 | -72.6131375431084 |
| 23 | 3.0 | 81.9101577198872 |
| 24 | 0.0 | -10.535815087014441 |
| 25 | 0.0 | 34.80417403574626 |
| 26 | 1.0 | -93.18054157849781 |
| 27 | 0.0 | -13.869213683784096 |
| 28 | 0.0 | -203.0626340350333 |
| 29 | 0.0 | -221.66542035133395 |
| 30 | 2.0 | -74.41141491271452 |
| 31 | 0.0 | -184.45997824560607 |
| 32 | 0.0 | -179.39267210537122 |
| 33 | 0.0 | -192.76142035133415 |
| 34 | 0.0 | -103.81518649068568 |
| 35 | 0.0 | 19.602190351605373 |
| 36 | 0.0 | 25.002862456891762 |
| 37 | 29.0 | 90.54477000035666 |
| 38 | 0.0 | 90.2787101755394 |
| 39 | 1.0 | -27.07122999964338 |
| 40 | 2.0 | 27.169496491839936 |
| 41 | 1.0 | -72.57778789391577 |
| 42 | 1.0 | 12.800780877596083 |
| 43 | 8.0 | -9.702481753680729 |
| 44 | 11.0 | 24.435491053220574 |
| 45 | 14.0 | 25.10215771988726 |
| 46 | 32.0 | -75.51384228011281 |
| 47 | 59.0 | -45.645289824460434 |
| 48 | 135.0 | 257.06137684220613 |
| 49 | 258.0 | 202.21479719345518 |
| 50 | 663.0 | 528.8792952629099 |
| 51 | 1662.0 | 1243.7471647360355 |
| 52 | 2729.0 | 2037.5355431558557 |
| 1 | 733.0 | 1517.4835214013788 |
| 2 | 1009.0 | 530.7850396477811 |
| 3 | 1157.0 | 1348.0487024533572 |
| 4 | 1454.0 | 1555.7765392947588 |
| 5 | 1134.0 | 1186.8865991195767 |
| 6 | 755.0 | 795.1172440317685 |
| 7 | 395.0 | 629.1980901728864 |
| 8 | 301.0 | 601.9742968404364 |
| 9 | 172.0 | 417.66303420916023 |
| 10 | 136.0 | 309.9457335075455 |
| 11 | 145.0 | 323.9271864905134 |
| 12 | 161.0 | 112.38514842017565 |
| 13 | 107.0 | 186.52470473691898 |
| 14 | 54.0 | 90.3328352637929 |
| 15 | 44.0 | 101.73677000035639 |
| 16 | 75.0 | 157.60538771944528 |
| 17 | 77.0 | 149.93875368449716 |
| 18 | 53.0 | -91.22271017570975 |
| 19 | 24.0 | 9.02800000000002 |
| 20 | 15.0 | 8.262135965408277 |
| 21 | 13.0 | -85.04912666586887 |
| 22 | 19.0 | -44.2091375431084 |
| 23 | 1.0 | 53.5061577198872 |
| 24 | 0.0 | 46.27218491298555 |
| 25 | 1.0 | 120.0161740357463 |
| 26 | 0.0 | 105.64745842150222 |
| 27 | 0.0 | 184.95878631621594 |
| 28 | 0.0 | 24.169365964966687 |
| 29 | 1.0 | -22.837420351333947 |
| 30 | 0.0 | -46.00741491271452 |
| 31 | 0.0 | 156.38802175439395 |
| 32 | 1.0 | 161.4553278946288 |
| 33 | 4.0 | 119.68257964866581 |
| 34 | 0.0 | -47.007186490685655 |
| 35 | 0.0 | 19.602190351605373 |
| 36 | 0.0 | 25.002862456891762 |
| 37 | 1.0 | -51.475229999643375 |
| 38 | 1.0 | -23.337289824460612 |
| 39 | 0.0 | 256.96877000035664 |
| 40 | 3.0 | -86.44650350816008 |
| 41 | 5.0 | 69.44221210608424 |
| 42 | 5.0 | 325.24478087759616 |
| 43 | 10.0 | 47.105518246319264 |
| 44 | 2.0 | -89.18050894677944 |
| 45 | 3.0 | 25.10215771988726 |
| 46 | 3.0 | 38.1021577198872 |
| 47 | 2.0 | 266.7987101755395 |
| 48 | 1.0 | 115.04137684220615 |
| 49 | 8.0 | 60.194797193455145 |
| 50 | 9.0 | 17.607295262909872 |
| 51 | 18.0 | 135.9911647360355 |
| 52 | 27.0 | 191.27554315585564 |
| 53 | 22.0 | 21.99884929609209 |
| 1 | 47.0 | -101.54447859862128 |
| 2 | 108.0 | 331.9570396477812 |
| 3 | 447.0 | 694.7567024533573 |
| 4 | 1067.0 | 1640.9885392947588 |
| 5 | 1728.0 | 1754.9665991195768 |
| 6 | 2224.0 | 1846.0652440317683 |
| 7 | 2197.0 | 1481.3180901728863 |
| 8 | 2143.0 | 1510.9022968404365 |
| 9 | 1973.0 | 1241.3790342091604 |
| 10 | 1290.0 | 934.8337335075455 |
| 11 | 798.0 | 607.9671864905135 |
| 12 | 444.0 | 254.40514842017564 |
| 13 | 237.0 | 129.71670473691898 |
| 14 | 103.0 | 147.1408352637929 |
| 15 | 56.0 | 16.524770000356398 |
| 16 | 47.0 | 43.98938771944523 |
| 17 | 23.0 | 93.13075368449711 |
| 18 | 13.0 | 107.60528982429025 |
| 19 | 7.0 | -76.184 |
| 20 | 7.0 | 150.28213596540832 |
| 21 | 2.0 | 56.97087333413111 |
| 22 | 1.0 | 126.21486245689158 |
| 23 | 2.0 | -3.301842280112794 |
| 24 | 0.0 | 17.868184912985555 |
| 25 | 0.0 | -50.40782596425373 |
| 26 | 0.0 | -7.96854157849782 |
| 27 | 0.0 | -42.27321368378409 |
| 28 | 1.0 | 52.57336596496668 |
| 29 | 1.0 | -22.837420351333947 |
| 30 | 0.0 | -74.41141491271452 |
| 31 | 0.0 | -42.439978245606056 |
| 32 | 0.0 | 303.47532789462883 |
| 33 | 1.0 | 34.47057964866582 |
| 34 | 0.0 | 66.6088135093143 |
| 35 | 0.0 | 48.00619035160537 |
| 36 | 1.0 | -60.20913754310823 |
| 37 | 1.0 | 175.75677000035665 |
| 38 | 0.0 | 33.47071017553941 |
| 39 | 0.0 | -27.07122999964338 |
| 40 | 1.0 | 112.38149649183993 |
| 41 | 8.0 | 126.25021210608423 |
| 42 | 4.0 | -44.00721912240388 |
| 43 | 1.0 | -66.51048175368072 |
| 44 | 8.0 | 52.83949105322057 |
| 45 | 14.0 | 167.12215771988724 |
| 46 | 48.0 | 180.12215771988718 |
| 47 | 81.0 | 11.16271017553953 |
| 48 | 89.0 | 29.82937684220616 |
| 49 | 89.0 | 202.21479719345518 |
| 50 | 160.0 | 273.24329526290984 |
| 51 | 298.0 | 476.83916473603546 |
| 52 | 230.0 | 390.1035431558557 |
| 1 | 246.0 | -16.33247859862132 |
| 2 | 530.0 | 275.1490396477812 |
| 3 | 1611.0 | 1291.2407024533572 |
| 4 | 2662.0 | 1811.4125392947587 |
| 5 | 2404.0 | 1783.3705991195768 |
| 6 | 1450.0 | 1107.5612440317684 |
| 7 | 1074.0 | 771.2180901728864 |
| 8 | 548.0 | 715.5902968404364 |
| 9 | 382.0 | 786.9150342091602 |
| 10 | 271.0 | 423.5617335075455 |
| 11 | 211.0 | 267.1191864905134 |
| 12 | 114.0 | 55.57714842017566 |
| 13 | 82.0 | 101.31270473691899 |
| 14 | 45.0 | 5.120835263792856 |
| 15 | 53.0 | -11.879229999643599 |
| 16 | 74.0 | 15.585387719445237 |
| 17 | 47.0 | 121.5347536844971 |
| 18 | 34.0 | -91.22271017570975 |
| 19 | 34.0 | 37.432000000000016 |
| 20 | 21.0 | -20.14186403459169 |
| 21 | 12.0 | 85.37487333413111 |
| 22 | 11.0 | 12.598862456891595 |
| 23 | 8.0 | -116.91784228011278 |
| 24 | 10.0 | -38.939815087014466 |
| 25 | 6.0 | -50.40782596425373 |
| 26 | 7.0 | -36.37254157849782 |
| 27 | 0.0 | -13.869213683784096 |
| 28 | 1.0 | 24.169365964966687 |
| 29 | 2.0 | 147.5865796486661 |
| 30 | 6.0 | -46.00741491271452 |
| 31 | 1.0 | -70.84397824560605 |
| 32 | 2.0 | 47.83932789462881 |
| 33 | 1.0 | -135.95342035133416 |
| 34 | 1.0 | -18.603186490685687 |
| 35 | 3.0 | -8.801809648394624 |
| 36 | 6.0 | 25.002862456891762 |
| 37 | 1.0 | -79.87922999964337 |
| 38 | 0.0 | -80.1452898244606 |
| 39 | 7.0 | -140.68722999964336 |
| 40 | 15.0 | -29.638503508160056 |
| 41 | 5.0 | -44.173787893915744 |
| 42 | 10.0 | -129.2192191224039 |
| 43 | 8.0 | -9.702481753680729 |
| 44 | 9.0 | -60.776508946779444 |
| 45 | 4.0 | -116.91784228011275 |
| 46 | 28.0 | -75.51384228011281 |
| 47 | 57.0 | -17.241289824460466 |
| 48 | 76.0 | -55.38262315779383 |
| 49 | 175.0 | 145.40679719345513 |
| 50 | 322.0 | 216.43529526290985 |
| 51 | 528.0 | 533.6471647360356 |
| 52 | 497.0 | 532.1235431558557 |
