# Supplementary material for: The relationship between seasonal influenza and telephone triage for fever: A population-based study in Osaka, Japan
Source: PLoS One. 2020 Aug 6;15(8):e0236560. doi: 10.1371/journal.pone.0236560 (PMC7410252; doi:10.1371/journal.pone.0236560)
Supplement: S1 File — (ZIP) [file pone.0236560.s001.zip › Age group/Figure 4_15-19 years old.pptx]

## Slide 1
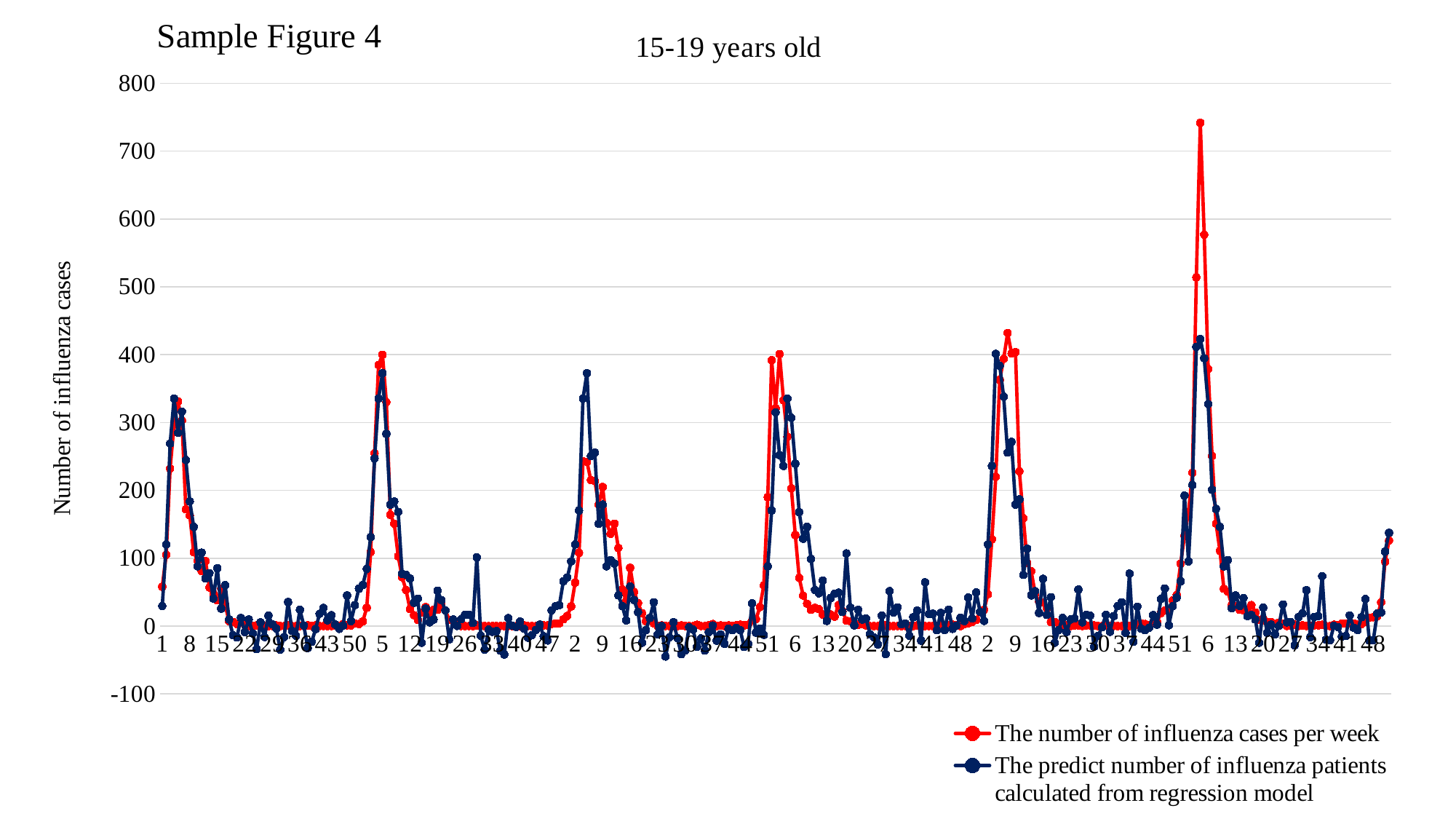

### Chart: 15-19 years old
| Category | The number of influenza cases per week | The predict number of influenza patients calculated from regression model |
|---|---|---|
| 1 | 58.0 | 29.51658856646314 |
| 2 | 105.0 | 120.2616544576963 |
| 3 | 232.0 | 268.9880151168144 |
| 4 | 297.0 | 335.39790775225725 |
| 5 | 331.0 | 284.9989350780422 |
| 6 | 303.0 | 316.2811478685726 |
| 7 | 172.0 | 244.77184089173298 |
| 8 | 163.0 | 183.66552073664624 |
| 9 | 109.0 | 146.20332015507128 |
| 10 | 96.0 | 88.04202732576965 |
| 11 | 81.0 | 108.27874670542306 |
| 12 | 96.0 | 70.16945387612154 |
| 13 | 57.0 | 78.10766666665143 |
| 14 | 48.0 | 40.48369302314483 |
| 15 | 38.0 | 85.20122693805331 |
| 16 | 55.0 | 25.70428062033165 |
| 17 | 31.0 | 60.26550658910825 |
| 18 | 7.0 | 9.690560271386673 |
| 19 | 6.0 | -13.603532945631677 |
| 20 | 3.0 | -16.76626647282339 |
| 21 | 7.0 | 11.993000000000002 |
| 22 | 2.0 | -8.801573449663405 |
| 23 | 0.0 | 9.650359689811545 |
| 24 | 0.0 | -10.80157344966338 |
| 25 | 1.0 | -33.91575988370004 |
| 26 | 0.0 | 5.48762616261984 |
| 27 | 0.0 | -16.12608003878677 |
| 28 | 0.0 | 15.467960465244985 |
| 29 | 0.0 | 2.4988540699800836 |
| 30 | 1.0 | -3.3226798449283876 |
| 31 | 0.0 | -34.74861298440336 |
| 32 | 0.0 | -16.292746705453425 |
| 33 | 1.0 | 35.420373837350006 |
| 34 | 0.0 | -7.147666666681754 |
| 35 | 0.0 | -14.296679844928384 |
| 36 | 0.0 | 23.945893604719963 |
| 37 | 0.0 | 0.834679844898254 |
| 38 | 1.0 | -32.419693023175014 |
| 39 | 0.0 | -23.27461298440336 |
| 40 | 1.0 | -4.318746705453421 |
| 41 | 0.0 | 18.129253294546608 |
| 42 | 0.0 | 26.774333333318253 |
| 43 | 0.0 | 8.483693023144902 |
| 44 | 0.0 | 16.133186434021574 |
| 45 | 1.0 | 2.1684934108616645 |
| 46 | 0.0 | -4.142773061946617 |
| 47 | 3.0 | 0.6856666666516347 |
| 48 | 1.0 | 45.08166666665161 |
| 49 | 1.0 | 7.3523333333183345 |
| 50 | 4.0 | 30.989066860510007 |
| 51 | 3.0 | 55.02878720945491 |
| 52 | 7.0 | 60.6293343026098 |
| 1 | 27.0 | 84.38658856646316 |
| 2 | 109.0 | 131.23565445769628 |
| 3 | 255.0 | 247.04001511681443 |
| 4 | 385.0 | 335.39790775225725 |
| 5 | 400.0 | 372.79093507804225 |
| 6 | 330.0 | 283.35914786857256 |
| 7 | 164.0 | 178.92784089173298 |
| 8 | 151.0 | 183.66552073664624 |
| 9 | 103.0 | 168.1513201550713 |
| 10 | 72.0 | 77.06802732576963 |
| 11 | 53.0 | 75.35674670542303 |
| 12 | 25.0 | 70.16945387612154 |
| 13 | 16.0 | 34.21166666665143 |
| 14 | 9.0 | 40.48369302314483 |
| 15 | 20.0 | -24.538773061946696 |
| 16 | 28.0 | 25.70428062033165 |
| 17 | 17.0 | 5.395506589108251 |
| 18 | 24.0 | 9.690560271386673 |
| 19 | 24.0 | 52.24046705436833 |
| 20 | 32.0 | 38.10373352717661 |
| 21 | 23.0 | 22.967000000000006 |
| 22 | 9.0 | -19.775573449663405 |
| 23 | 7.0 | 9.650359689811545 |
| 24 | 4.0 | 0.1724265503366169 |
| 25 | 0.0 | 9.98024011629996 |
| 26 | 0.0 | 16.461626162619844 |
| 27 | 0.0 | 16.795919961213237 |
| 28 | 0.0 | 4.493960465244982 |
| 29 | 1.0 | 101.26485406998009 |
| 30 | 0.0 | -14.296679844928391 |
| 31 | 0.0 | -34.74861298440336 |
| 32 | 0.0 | -5.318746705453428 |
| 33 | 0.0 | -8.475626162649988 |
| 34 | 0.0 | -7.147666666681754 |
| 35 | 0.0 | -36.24467984492838 |
| 36 | 0.0 | -41.89810639528004 |
| 37 | 0.0 | 11.808679844898258 |
| 38 | 0.0 | 0.5023069768249897 |
| 39 | 0.0 | -1.3266129844033507 |
| 40 | 0.0 | 6.655253294546583 |
| 41 | 1.0 | -3.8187467054533997 |
| 42 | 0.0 | -17.12166666668174 |
| 43 | 0.0 | -13.464306976855092 |
| 44 | 0.0 | -5.814813565978426 |
| 45 | 1.0 | 2.1684934108616645 |
| 46 | 1.0 | -15.11677306194662 |
| 47 | 1.0 | -21.26233333334836 |
| 48 | 3.0 | 23.133666666651614 |
| 49 | 4.0 | 29.300333333318342 |
| 50 | 4.0 | 30.989066860510007 |
| 51 | 10.0 | 66.00278720945491 |
| 52 | 15.0 | 71.6033343026098 |
| 1 | 29.0 | 95.36058856646315 |
| 2 | 64.0 | 120.2616544576963 |
| 3 | 108.0 | 170.22201511681445 |
| 4 | 243.0 | 335.39790775225725 |
| 5 | 242.0 | 372.79093507804225 |
| 6 | 215.0 | 250.43714786857262 |
| 7 | 214.0 | 255.74584089173297 |
| 8 | 179.0 | 150.74352073664627 |
| 9 | 205.0 | 179.12532015507128 |
| 10 | 152.0 | 88.04202732576965 |
| 11 | 136.0 | 97.30474670542307 |
| 12 | 151.0 | 92.11745387612154 |
| 13 | 115.0 | 45.185666666651436 |
| 14 | 54.0 | 29.509693023144834 |
| 15 | 37.0 | 8.383226938053305 |
| 16 | 86.0 | 58.62628062033165 |
| 17 | 50.0 | 38.317506589108255 |
| 18 | 34.0 | 20.664560271386677 |
| 19 | 19.0 | -24.577532945631674 |
| 20 | 7.0 | -5.792266472823393 |
| 21 | 10.0 | 11.993000000000002 |
| 22 | 4.0 | 35.094426550336586 |
| 23 | 0.0 | -12.297640310188456 |
| 24 | 1.0 | 0.1724265503366169 |
| 25 | 0.0 | -44.889759883700044 |
| 26 | 0.0 | -16.460373837380157 |
| 27 | 0.0 | 5.821919961213233 |
| 28 | 0.0 | -17.45403953475501 |
| 29 | 1.0 | -41.39714593001992 |
| 30 | 0.0 | -36.244679844928385 |
| 31 | 0.0 | -1.8266129844033543 |
| 32 | 0.0 | -5.318746705453428 |
| 33 | 2.0 | -30.423626162649995 |
| 34 | 0.0 | -18.12166666668175 |
| 35 | 0.0 | -36.24467984492838 |
| 36 | 1.0 | -8.976106395280038 |
| 37 | 3.0 | 0.834679844898254 |
| 38 | 0.0 | -21.445693023175018 |
| 39 | 1.0 | -12.300612984403354 |
| 40 | 0.0 | -26.266746705453414 |
| 41 | 1.0 | -3.8187467054533997 |
| 42 | 0.0 | -6.147666666681744 |
| 43 | 1.0 | -2.490306976855095 |
| 44 | 2.0 | -5.814813565978426 |
| 45 | 1.0 | -30.753506589138336 |
| 46 | 2.0 | -26.090773061946617 |
| 47 | 8.0 | 33.60766666665163 |
| 48 | 10.0 | -9.788333333348387 |
| 49 | 28.0 | -3.6216666666816657 |
| 50 | 60.0 | -12.90693313949 |
| 51 | 190.0 | 87.95078720945492 |
| 52 | 392.0 | 170.36933430260981 |
| 1 | 321.0 | 314.8405885664631 |
| 2 | 401.0 | 251.9496544576963 |
| 3 | 333.0 | 236.06601511681444 |
| 4 | 279.0 | 335.39790775225725 |
| 5 | 203.0 | 306.9469350780422 |
| 6 | 134.0 | 239.46314786857263 |
| 7 | 71.0 | 167.95384089173297 |
| 8 | 45.0 | 128.79552073664627 |
| 9 | 33.0 | 146.20332015507128 |
| 10 | 24.0 | 99.01602732576964 |
| 11 | 27.0 | 53.40874670542305 |
| 12 | 25.0 | 48.22145387612155 |
| 13 | 17.0 | 67.13366666665144 |
| 14 | 16.0 | 7.561693023144834 |
| 15 | 17.0 | 41.3052269380533 |
| 16 | 14.0 | 47.652280620331645 |
| 17 | 31.0 | 49.29150658910826 |
| 18 | 22.0 | 20.664560271386677 |
| 19 | 8.0 | 107.11046705436834 |
| 20 | 7.0 | 27.129733527176604 |
| 21 | 3.0 | 1.0189999999999984 |
| 22 | 2.0 | 24.120426550336596 |
| 23 | 3.0 | 9.650359689811545 |
| 24 | 1.0 | 11.14642655033662 |
| 25 | 0.0 | -11.967759883700047 |
| 26 | 0.0 | -16.460373837380157 |
| 27 | 0.0 | -27.10008003878677 |
| 28 | 2.0 | 15.467960465244985 |
| 29 | 1.0 | -41.39714593001992 |
| 30 | 0.0 | 51.54732015507161 |
| 31 | 0.0 | 20.12138701559664 |
| 32 | 0.0 | 27.60325329454657 |
| 33 | 0.0 | 2.4983738373500017 |
| 34 | 0.0 | 3.826333333318253 |
| 35 | 0.0 | -14.296679844928384 |
| 36 | 0.0 | 12.97189360471996 |
| 37 | 1.0 | 22.78267984489826 |
| 38 | 0.0 | -21.445693023175018 |
| 39 | 0.0 | 64.51738701559665 |
| 40 | 0.0 | 17.629253294546587 |
| 41 | 0.0 | 18.129253294546608 |
| 42 | 0.0 | -6.147666666681744 |
| 43 | 1.0 | 19.457693023144905 |
| 44 | 0.0 | -5.814813565978426 |
| 45 | 1.0 | 24.116493410861658 |
| 46 | 2.0 | -4.142773061946617 |
| 47 | 1.0 | 0.6856666666516347 |
| 48 | 0.0 | 12.15966666665161 |
| 49 | 3.0 | 7.3523333333183345 |
| 50 | 4.0 | 41.96306686050999 |
| 51 | 6.0 | 11.13278720945491 |
| 52 | 9.0 | 49.655334302609795 |
| 53 | 21.0 | 21.00518837260517 |
| 1 | 24.0 | 7.568588566463134 |
| 2 | 47.0 | 120.2616544576963 |
| 3 | 128.0 | 236.06601511681444 |
| 4 | 220.0 | 401.24190775225725 |
| 5 | 363.0 | 383.7649350780422 |
| 6 | 394.0 | 338.22914786857257 |
| 7 | 432.0 | 255.74584089173297 |
| 8 | 402.0 | 271.45752073664625 |
| 9 | 404.0 | 179.12532015507128 |
| 10 | 228.0 | 186.80802732576964 |
| 11 | 159.0 | 75.35674670542303 |
| 12 | 93.0 | 114.06545387612155 |
| 13 | 81.0 | 45.185666666651436 |
| 14 | 48.0 | 51.457693023144834 |
| 15 | 36.0 | 19.35722693805331 |
| 16 | 35.0 | 69.60028062033166 |
| 17 | 26.0 | 16.369506589108248 |
| 18 | 6.0 | 42.61256027138666 |
| 19 | 6.0 | -24.577532945631674 |
| 20 | 4.0 | -5.792266472823393 |
| 21 | 1.0 | 11.993000000000002 |
| 22 | 1.0 | -8.801573449663405 |
| 23 | 0.0 | 9.650359689811545 |
| 24 | 1.0 | 11.14642655033662 |
| 25 | 1.0 | 53.87624011629996 |
| 26 | 0.0 | 5.48762616261984 |
| 27 | 1.0 | 16.795919961213237 |
| 28 | 1.0 | 15.467960465244985 |
| 29 | 1.0 | -30.423145930019913 |
| 30 | 0.0 | -14.296679844928391 |
| 31 | 0.0 | -1.8266129844033543 |
| 32 | 1.0 | 16.62925329454658 |
| 33 | 0.0 | -8.475626162649988 |
| 34 | 0.0 | 14.800333333318257 |
| 35 | 0.0 | 29.599320155071617 |
| 36 | 0.0 | 34.91989360471997 |
| 37 | 1.0 | -10.13932015510174 |
| 38 | 0.0 | 77.32030697682498 |
| 39 | 1.0 | -23.27461298440336 |
| 40 | 2.0 | 28.603253294546576 |
| 41 | 4.0 | -3.8187467054533997 |
| 42 | 3.0 | -6.147666666681744 |
| 43 | 1.0 | -2.490306976855095 |
| 44 | 5.0 | 16.133186434021574 |
| 45 | 6.0 | 2.1684934108616645 |
| 46 | 19.0 | 39.75322693805338 |
| 47 | 23.0 | 55.55566666665164 |
| 48 | 24.0 | 1.1856666666516062 |
| 49 | 38.0 | 29.300333333318342 |
| 50 | 46.0 | 41.96306686050999 |
| 51 | 92.0 | 66.00278720945491 |
| 52 | 133.0 | 192.31733430260982 |
| 1 | 168.0 | 95.36058856646315 |
| 2 | 226.0 | 208.0536544576963 |
| 3 | 514.0 | 411.6500151168144 |
| 4 | 742.0 | 423.1899077522572 |
| 5 | 577.0 | 394.73893507804223 |
| 6 | 379.0 | 327.2551478685726 |
| 7 | 251.0 | 200.87584089173296 |
| 8 | 151.0 | 172.69152073664625 |
| 9 | 111.0 | 146.20332015507128 |
| 10 | 55.0 | 88.04202732576965 |
| 11 | 51.0 | 97.30474670542307 |
| 12 | 31.0 | 26.27345387612155 |
| 13 | 29.0 | 45.185666666651436 |
| 14 | 24.0 | 29.509693023144834 |
| 15 | 23.0 | 41.3052269380533 |
| 16 | 24.0 | 14.730280620331648 |
| 17 | 31.0 | 16.369506589108248 |
| 18 | 20.0 | 9.690560271386673 |
| 19 | 9.0 | -24.577532945631674 |
| 20 | 11.0 | 27.129733527176604 |
| 21 | 6.0 | -9.954999999999998 |
| 22 | 6.0 | 2.1724265503365885 |
| 23 | 4.0 | -12.297640310188456 |
| 24 | 5.0 | 0.1724265503366169 |
| 25 | 3.0 | 31.928240116299953 |
| 26 | 0.0 | 5.48762616261984 |
| 27 | 1.0 | 5.821919961213233 |
| 28 | 2.0 | -28.428039534755015 |
| 29 | 0.0 | 13.472854069980087 |
| 30 | 1.0 | 18.625320155071606 |
| 31 | 0.0 | 53.04338701559666 |
| 32 | 0.0 | -16.292746705453425 |
| 33 | 1.0 | 13.472373837350005 |
| 34 | 1.0 | 14.800333333318257 |
| 35 | 2.0 | 73.49532015507162 |
| 36 | 0.0 | -19.95010639528004 |
| 37 | 0.0 | -21.11332015510174 |
| 38 | 2.0 | 0.5023069768249897 |
| 39 | 1.0 | -1.3266129844033507 |
| 40 | 4.0 | -15.292746705453418 |
| 41 | 4.0 | -14.792746705453396 |
| 42 | 4.0 | 15.800333333318264 |
| 43 | 4.0 | -2.490306976855095 |
| 44 | 2.0 | -5.814813565978426 |
| 45 | 3.0 | 13.142493410861668 |
| 46 | 6.0 | 39.75322693805338 |
| 47 | 12.0 | -21.26233333334836 |
| 48 | 13.0 | -20.762333333348387 |
| 49 | 14.0 | 18.32633333331834 |
| 50 | 35.0 | 20.015066860510004 |
| 51 | 95.0 | 109.89878720945492 |
| 52 | 126.0 | 137.44733430260982 |
