# Supplementary material for: The relationship between seasonal influenza and telephone triage for fever: A population-based study in Osaka, Japan
Source: PLoS One. 2020 Aug 6;15(8):e0236560. doi: 10.1371/journal.pone.0236560 (PMC7410252; doi:10.1371/journal.pone.0236560)
Supplement: S1 File — (ZIP) [file pone.0236560.s001.zip › Age group/Figure 4_5-9 years old.pptx]

## Slide 1
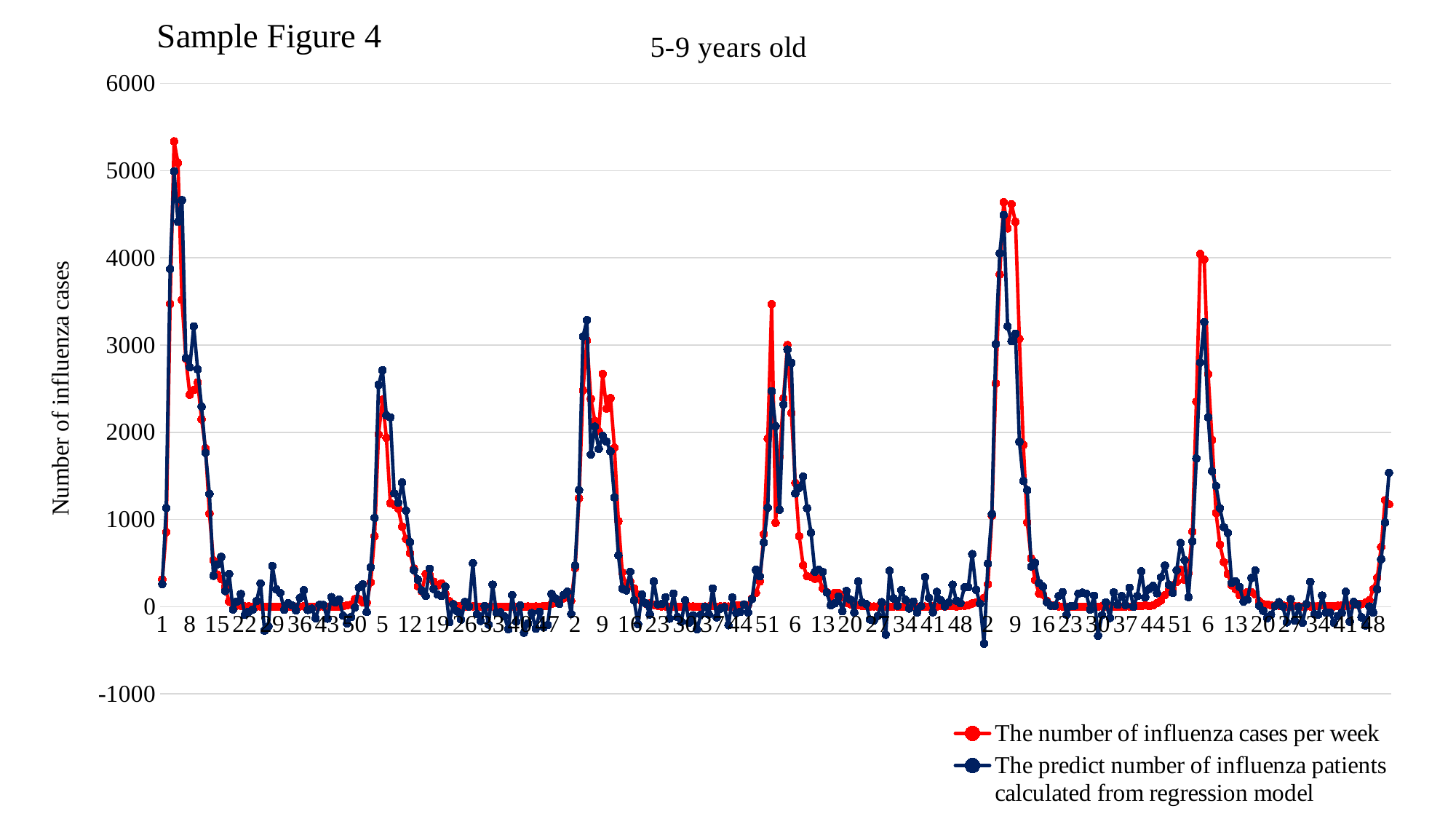

### Chart: 5-9 years old
| Category | The number of influenza cases per week | The predict number of influenza patients calculated from regression model |
|---|---|---|
| 1 | 311.0 | 257.7988855810468 |
| 2 | 853.0 | 1132.0574048468347 |
| 3 | 3472.0 | 3871.0734795582453 |
| 4 | 5335.0 | 4991.840692751048 |
| 5 | 5089.0 | 4413.8022461747205 |
| 6 | 3517.0 | 4661.09350109693 |
| 7 | 2841.0 | 2851.6434637412226 |
| 8 | 2430.0 | 2746.1302455007376 |
| 9 | 2484.0 | 3214.3475263353266 |
| 10 | 2571.0 | 2721.160764766528 |
| 11 | 2147.0 | 2292.646748949507 |
| 12 | 1816.0 | 1763.150643952018 |
| 13 | 1066.0 | 1291.3726799597594 |
| 14 | 531.0 | 353.98979252876944 |
| 15 | 366.0 | 484.0139500250062 |
| 16 | 314.0 | 569.972175837009 |
| 17 | 224.0 | 179.10255914524802 |
| 18 | 59.0 | 373.2055515737294 |
| 19 | 30.0 | -32.264999999999986 |
| 20 | 12.0 | 58.45818088468826 |
| 21 | 12.0 | 144.23214420296546 |
| 22 | 3.0 | -93.19517331334603 |
| 23 | 3.0 | -57.571855797034615 |
| 24 | 1.0 | -33.686070839694736 |
| 25 | 2.0 | 65.65740417285251 |
| 26 | 3.0 | 267.2770474509782 |
| 27 | 0.0 | -275.9001283861191 |
| 28 | 0.0 | -225.39206511803144 |
| 29 | 0.0 | 466.3052056211982 |
| 30 | 0.0 | 198.86824987443998 |
| 31 | 1.0 | 158.28941494219478 |
| 32 | 0.0 | -34.46956671694335 |
| 33 | 0.0 | 39.70525744595912 |
| 34 | 0.0 | 15.531881709238618 |
| 35 | 1.0 | -42.61813410778373 |
| 36 | 2.0 | 101.84991839096135 |
| 37 | 4.0 | 188.33714167912584 |
| 38 | 0.0 | -37.68512586227979 |
| 39 | 0.0 | -21.99585579703505 |
| 40 | 1.0 | -133.5330107694311 |
| 41 | 2.0 | 21.581404172851435 |
| 42 | 0.0 | 20.032196701710134 |
| 43 | 0.0 | -137.46160912033082 |
| 44 | 0.0 | 110.01658505762819 |
| 45 | 0.0 | 47.93860087465027 |
| 46 | 2.0 | 81.60851169418164 |
| 47 | 5.0 | -103.93752498754135 |
| 48 | 14.0 | -194.80779252894712 |
| 49 | 24.0 | -119.30486336855346 |
| 50 | 86.0 | -9.834585057805441 |
| 51 | 87.0 | 219.2803865059736 |
| 52 | 49.0 | 256.52455477155195 |
| 1 | 44.0 | -61.52111441895312 |
| 2 | 279.0 | 450.8414048468347 |
| 3 | 809.0 | 1018.4814795582453 |
| 4 | 1974.0 | 2543.720692751048 |
| 5 | 2373.0 | 2710.7622461747205 |
| 6 | 1935.0 | 2191.685501096929 |
| 7 | 1187.0 | 2170.4274637412227 |
| 8 | 1169.0 | 1298.5462455007378 |
| 9 | 1124.0 | 1191.987526335327 |
| 10 | 917.0 | 1422.5927647665283 |
| 11 | 776.0 | 1100.518748949507 |
| 12 | 614.0 | 741.3266439520182 |
| 13 | 437.0 | 418.5646799597594 |
| 14 | 236.0 | 311.4137925287694 |
| 15 | 175.0 | 185.98195002500614 |
| 16 | 377.0 | 122.92417583700896 |
| 17 | 361.0 | 434.55855914524807 |
| 18 | 286.0 | 202.90155157372928 |
| 19 | 241.0 | 138.039 |
| 20 | 266.0 | 122.3221808846883 |
| 21 | 148.0 | 229.3841442029654 |
| 22 | 65.0 | -178.34717331334608 |
| 23 | 29.0 | 27.58014420296537 |
| 24 | 8.0 | -54.97407083969475 |
| 25 | 9.0 | -147.22259582714742 |
| 26 | 0.0 | 54.39704745097819 |
| 27 | 2.0 | 0.8438716138809355 |
| 28 | 3.0 | 498.3999348819686 |
| 29 | 2.0 | -87.18279437880153 |
| 30 | 1.0 | -163.0277501255601 |
| 31 | 0.0 | 9.273414942194819 |
| 32 | 0.0 | -204.77356671694332 |
| 33 | 0.0 | 252.58525744595912 |
| 34 | 0.0 | -69.62011829076137 |
| 35 | 0.0 | -63.90613410778374 |
| 36 | 0.0 | -111.03008160903865 |
| 37 | 0.0 | -258.7108583208742 |
| 38 | 2.0 | 132.6188741377203 |
| 39 | 0.0 | -171.01185579703503 |
| 40 | 0.0 | 15.482989230568947 |
| 41 | 1.0 | -297.73859582714863 |
| 42 | 2.0 | -192.84780329828988 |
| 43 | 1.0 | -73.59760912033086 |
| 44 | 2.0 | -251.87941494237182 |
| 45 | 0.0 | -58.50139912534973 |
| 46 | 6.0 | -237.7114883058184 |
| 47 | 6.0 | -210.37752498754134 |
| 48 | 33.0 | 145.8002074710529 |
| 49 | 44.0 | 93.57513663144648 |
| 50 | 39.0 | 32.741414942194524 |
| 51 | 94.0 | 134.12838650597368 |
| 52 | 115.0 | 171.37255477155202 |
| 1 | 66.0 | -82.80911441895313 |
| 2 | 442.0 | 472.1294048468347 |
| 3 | 1243.0 | 1337.8014795582453 |
| 4 | 2479.0 | 3097.208692751048 |
| 5 | 3051.0 | 3285.5382461747204 |
| 6 | 2382.0 | 1744.637501096929 |
| 7 | 2131.0 | 2063.9874637412227 |
| 8 | 2011.0 | 1809.4582455007378 |
| 9 | 2667.0 | 1958.3555263353269 |
| 10 | 2270.0 | 1890.9287647665283 |
| 11 | 2390.0 | 1781.7347489495069 |
| 12 | 1823.0 | 1252.2386439520183 |
| 13 | 980.0 | 588.8686799597594 |
| 14 | 392.0 | 204.97379252876942 |
| 15 | 241.0 | 185.98195002500614 |
| 16 | 289.0 | 399.668175837009 |
| 17 | 210.0 | 72.66255914524808 |
| 18 | 138.0 | -201.5704484262707 |
| 19 | 60.0 | 138.039 |
| 20 | 34.0 | 37.17018088468831 |
| 21 | 38.0 | -89.9358557970346 |
| 22 | 21.0 | 289.988826686654 |
| 23 | 11.0 | 27.58014420296537 |
| 24 | 3.0 | 30.177929160305183 |
| 25 | 3.0 | 108.23340417285253 |
| 26 | 0.0 | -137.1949525490218 |
| 27 | 0.0 | 149.8598716138809 |
| 28 | 0.0 | -118.95206511803138 |
| 29 | 0.0 | -172.33479437880158 |
| 30 | 0.0 | 71.14024987443992 |
| 31 | 2.0 | -182.31858505780517 |
| 32 | 2.0 | -98.33356671694338 |
| 33 | 0.0 | -258.32674255404095 |
| 34 | 1.0 | -90.90811829076138 |
| 35 | 2.0 | -0.04213410778370985 |
| 36 | 2.0 | -89.74208160903869 |
| 37 | 10.0 | 209.62514167912585 |
| 38 | 1.0 | -122.83712586227972 |
| 39 | 3.0 | -21.99585579703505 |
| 40 | 4.0 | -5.805010769431064 |
| 41 | 2.0 | -212.58659582714864 |
| 42 | 4.0 | 105.18419670171018 |
| 43 | 15.0 | -73.59760912033086 |
| 44 | 15.0 | -60.28741494237184 |
| 45 | 22.0 | 26.65060087465026 |
| 46 | 18.0 | -67.40748830581838 |
| 47 | 95.0 | 87.65447501245869 |
| 48 | 157.0 | 422.5442074710529 |
| 49 | 290.0 | 349.0311366314465 |
| 50 | 831.0 | 735.2454149421947 |
| 51 | 1925.0 | 1134.6643865059739 |
| 52 | 3468.0 | 2470.476554771552 |
| 1 | 961.0 | 2067.278885581047 |
| 2 | 1718.0 | 1110.7694048468347 |
| 3 | 2390.0 | 2317.0494795582454 |
| 4 | 2999.0 | 2948.1926927510476 |
| 5 | 2219.0 | 2795.9142461747206 |
| 6 | 1418.0 | 1297.589501096929 |
| 7 | 808.0 | 1361.4834637412227 |
| 8 | 474.0 | 1490.1382455007379 |
| 9 | 351.0 | 1128.123526335327 |
| 10 | 344.0 | 847.8167647665282 |
| 11 | 319.0 | 398.01474894950695 |
| 12 | 326.0 | 422.0066439520181 |
| 13 | 208.0 | 397.2766799597594 |
| 14 | 166.0 | 162.3977925287694 |
| 15 | 118.0 | 15.677950025006112 |
| 16 | 156.0 | 37.77217583700897 |
| 17 | 155.0 | 115.2385591452481 |
| 18 | 107.0 | -52.55444842627074 |
| 19 | 46.0 | 180.615 |
| 20 | 29.0 | 79.74618088468827 |
| 21 | 25.0 | -68.6478557970346 |
| 22 | 14.0 | 289.988826686654 |
| 23 | 6.0 | 48.86814420296538 |
| 24 | 7.0 | 30.177929160305183 |
| 25 | 2.0 | -147.22259582714742 |
| 26 | 2.0 | -158.4829525490218 |
| 27 | 2.0 | -105.59612838611912 |
| 28 | 1.0 | 51.35193488196859 |
| 29 | 3.0 | -321.35079437880165 |
| 30 | 0.0 | 411.74824987443986 |
| 31 | 2.0 | 94.42541494219475 |
| 32 | 1.0 | 8.106433283056674 |
| 33 | 0.0 | 188.72125744595908 |
| 34 | 0.0 | 79.39588170923865 |
| 35 | 0.0 | -21.33013410778372 |
| 36 | 3.0 | 59.27391839096133 |
| 37 | 0.0 | -67.11885832087418 |
| 38 | 0.0 | 4.890874137720232 |
| 39 | 2.0 | 339.900144202965 |
| 40 | 0.0 | 100.63498923056893 |
| 41 | 4.0 | -63.57059582714861 |
| 42 | 1.0 | 169.04819670171008 |
| 43 | 5.0 | 75.41839087966916 |
| 44 | 2.0 | 3.5765850576281935 |
| 45 | 5.0 | 47.93860087465027 |
| 46 | 7.0 | 251.91251169418163 |
| 47 | 2.0 | 66.36647501245868 |
| 48 | 10.0 | 39.360207471052824 |
| 49 | 10.0 | 221.30313663144653 |
| 50 | 18.0 | 224.3334149421945 |
| 51 | 36.0 | 602.4643865059737 |
| 52 | 47.0 | 192.66055477155203 |
| 53 | 35.0 | 35.01841309233839 |
| 1 | 100.0 | -423.4171144189531 |
| 2 | 256.0 | 493.4174048468347 |
| 3 | 1041.0 | 1061.0574795582452 |
| 4 | 2561.0 | 3012.056692751048 |
| 5 | 3808.0 | 4051.9062461747208 |
| 6 | 4637.0 | 4490.78950109693 |
| 7 | 4336.0 | 3213.539463741223 |
| 8 | 4615.0 | 3044.1622455007378 |
| 9 | 4413.0 | 3129.1955263353266 |
| 10 | 3072.0 | 1890.9287647665283 |
| 11 | 1853.0 | 1441.126748949507 |
| 12 | 966.0 | 1337.3906439520183 |
| 13 | 554.0 | 461.14067995975944 |
| 14 | 305.0 | 503.0057925287694 |
| 15 | 150.0 | 271.1339500250062 |
| 16 | 137.0 | 229.36417583700901 |
| 17 | 64.0 | 51.37455914524807 |
| 18 | 18.0 | 11.309551573729294 |
| 19 | 6.0 | 10.311000000000035 |
| 20 | 3.0 | 122.3221808846883 |
| 21 | 0.0 | 165.52014420296535 |
| 22 | 1.0 | -93.19517331334603 |
| 23 | 0.0 | 6.2921442029653605 |
| 24 | 2.0 | 8.889929160305172 |
| 25 | 0.0 | 150.80940417285257 |
| 26 | 0.0 | 160.83704745097813 |
| 27 | 0.0 | 149.8598716138809 |
| 28 | 0.0 | -33.800065118031455 |
| 29 | 0.0 | 125.69720562119835 |
| 30 | 0.0 | -333.3317501255602 |
| 31 | 4.0 | -97.16658505780524 |
| 32 | 0.0 | 50.682433283056696 |
| 33 | 0.0 | -130.59874255404097 |
| 34 | 0.0 | 164.5478817092386 |
| 35 | 1.0 | 21.245865892216244 |
| 36 | 1.0 | 123.13791839096136 |
| 37 | 1.0 | 18.033141679125855 |
| 38 | 1.0 | 217.77087413772023 |
| 39 | 1.0 | -0.7078557970350374 |
| 40 | 6.0 | 121.92298923056894 |
| 41 | 5.0 | 404.7654041728514 |
| 42 | 14.0 | 105.18419670171018 |
| 43 | 5.0 | 203.14639087966913 |
| 44 | 13.0 | 237.74458505762814 |
| 45 | 40.0 | 154.3786008746502 |
| 46 | 71.0 | 337.06451169418165 |
| 47 | 130.0 | 470.8384750124587 |
| 48 | 181.0 | 252.24020747105294 |
| 49 | 203.0 | 157.4391366314465 |
| 50 | 284.0 | 415.9254149421945 |
| 51 | 423.0 | 730.1923865059738 |
| 52 | 306.0 | 533.268554771552 |
| 1 | 384.0 | 108.78288558104697 |
| 2 | 860.0 | 748.8734048468348 |
| 3 | 2350.0 | 1699.697479558245 |
| 4 | 4044.0 | 2799.176692751048 |
| 5 | 3982.0 | 3264.250246174721 |
| 6 | 2667.0 | 2170.3975010969293 |
| 7 | 1911.0 | 1553.0754637412228 |
| 8 | 1073.0 | 1383.6982455007378 |
| 9 | 711.0 | 1128.123526335327 |
| 10 | 511.0 | 911.6807647665282 |
| 11 | 374.0 | 845.062748949507 |
| 12 | 244.0 | 272.99064395201816 |
| 13 | 203.0 | 290.8366799597594 |
| 14 | 132.0 | 226.26179252876938 |
| 15 | 151.0 | 58.253950025006134 |
| 16 | 167.0 | 80.348175837009 |
| 17 | 167.0 | 328.1185591452481 |
| 18 | 141.0 | 415.7815515737294 |
| 19 | 62.0 | 10.311000000000035 |
| 20 | 28.0 | -47.98181911531174 |
| 21 | 25.0 | -132.51185579703457 |
| 22 | 18.0 | -93.19517331334603 |
| 23 | 10.0 | 6.2921442029653605 |
| 24 | 11.0 | 51.465929160305194 |
| 25 | 16.0 | 1.7934041728525898 |
| 26 | 2.0 | -179.7709525490218 |
| 27 | 1.0 | 85.99587161388098 |
| 28 | 6.0 | -161.5280651180314 |
| 29 | 4.0 | -2.0307943788017155 |
| 30 | 0.0 | -184.3157501255601 |
| 31 | 4.0 | 30.56141494219483 |
| 32 | 3.0 | 284.8504332830567 |
| 33 | 4.0 | -88.02274255404095 |
| 34 | 7.0 | -90.90811829076138 |
| 35 | 17.0 | 127.6858658922163 |
| 36 | 7.0 | -68.45408160903868 |
| 37 | 8.0 | -67.11885832087418 |
| 38 | 4.0 | -186.70112586227975 |
| 39 | 11.0 | -107.14785579703504 |
| 40 | 18.0 | -69.6690107694311 |
| 41 | 9.0 | 170.59740417285138 |
| 42 | 14.0 | -171.55980329828986 |
| 43 | 22.0 | 54.13039087966915 |
| 44 | 32.0 | 24.864585057628148 |
| 45 | 29.0 | -122.36539912534977 |
| 46 | 45.0 | -216.42348830581838 |
| 47 | 75.0 | 2.5024750124586497 |
| 48 | 203.0 | -67.07979252894711 |
| 49 | 331.0 | 200.01513663144652 |
| 50 | 684.0 | 543.6534149421946 |
| 51 | 1220.0 | 964.3603865059737 |
| 52 | 1173.0 | 1533.804554771552 |
