# Supplementary material for: The relationship between seasonal influenza and telephone triage for fever: A population-based study in Osaka, Japan
Source: PLoS One. 2020 Aug 6;15(8):e0236560. doi: 10.1371/journal.pone.0236560 (PMC7410252; doi:10.1371/journal.pone.0236560)
Supplement: S1 File — (ZIP) [file pone.0236560.s001.zip › Age group/Figure 4_over20 years old.pptx]

## Slide 1
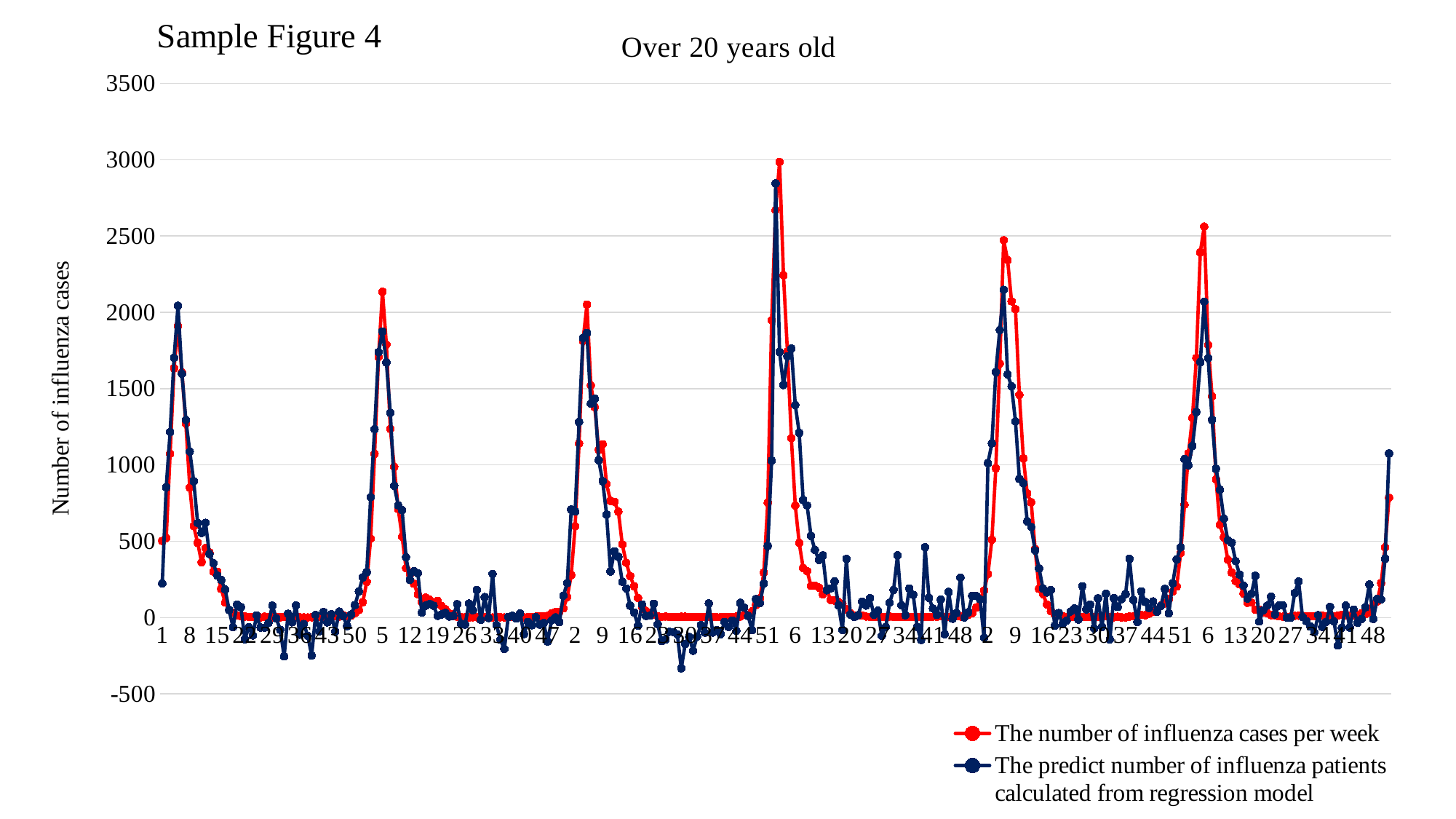

### Chart: Over 20 years old
| Category | The number of influenza cases per week | The predict number of influenza patients calculated from regression model |
|---|---|---|
| 1 | 502.0 | 222.7089725304943 |
| 2 | 521.0 | 853.6082560417904 |
| 3 | 1074.0 | 1215.7257767049678 |
| 4 | 1633.0 | 1701.69324391613 |
| 5 | 1911.0 | 2042.632747504268 |
| 6 | 1606.0 | 1596.773272209333 |
| 7 | 1270.0 | 1294.3305732075778 |
| 8 | 852.0 | 1086.6631199370631 |
| 9 | 598.0 | 893.4063454588736 |
| 10 | 490.0 | 619.1550040417694 |
| 11 | 363.0 | 554.1056877834758 |
| 12 | 455.0 | 620.6123243419506 |
| 13 | 427.0 | 416.4021168026752 |
| 14 | 301.0 | 355.93145417789503 |
| 15 | 301.0 | 275.1992385548474 |
| 16 | 188.0 | 245.9176293820817 |
| 17 | 98.0 | 183.14895463164294 |
| 18 | 50.0 | 49.642069619444726 |
| 19 | 33.0 | -63.041 |
| 20 | 14.0 | 85.59750540301366 |
| 21 | 9.0 | 68.37863028957673 |
| 22 | 7.0 | -142.527240781974 |
| 23 | 5.0 | -63.12020349750401 |
| 24 | 4.0 | -117.31944114497168 |
| 25 | 3.0 | 15.061700816630719 |
| 26 | 1.0 | -65.06662039104418 |
| 27 | 6.0 | -67.17674527760732 |
| 28 | 3.0 | -35.74656198964868 |
| 29 | 1.0 | 78.26282388820493 |
| 30 | 5.0 | -5.20092543117471 |
| 31 | 2.0 | -80.1106464573503 |
| 32 | 5.0 | -254.63771789189892 |
| 33 | 5.0 | 24.73310690391486 |
| 34 | 2.0 | -30.916082652826958 |
| 35 | 2.0 | 78.59010871890382 |
| 36 | 2.0 | -112.21464464236129 |
| 37 | 1.0 | -42.30069495998396 |
| 38 | 1.0 | -118.6516747505533 |
| 39 | 2.0 | -248.58908356032128 |
| 40 | 3.0 | 17.841621298309917 |
| 41 | 7.0 | -89.61005749401522 |
| 42 | 4.0 | 37.05165949027463 |
| 43 | 4.0 | -31.305198548122803 |
| 44 | 9.0 | 21.784909263401005 |
| 45 | 10.0 | -93.15683287970023 |
| 46 | 6.0 | 39.00405930877571 |
| 47 | 16.0 | 10.856375567069904 |
| 48 | 10.0 | -55.71612397918295 |
| 49 | 13.0 | 19.816067392548007 |
| 50 | 29.0 | 81.64764645712148 |
| 51 | 49.0 | 170.96020440476923 |
| 52 | 101.0 | 263.47226412556597 |
| 1 | 234.0 | 297.34097253049424 |
| 2 | 517.0 | 788.3052560417905 |
| 3 | 1073.0 | 1234.3837767049677 |
| 4 | 1707.0 | 1739.0092439161303 |
| 5 | 2135.0 | 1874.710747504268 |
| 6 | 1789.0 | 1671.405272209333 |
| 7 | 1236.0 | 1340.9755732075778 |
| 8 | 987.0 | 862.7671199370632 |
| 9 | 712.0 | 734.8133454588735 |
| 10 | 530.0 | 703.1160040417694 |
| 11 | 323.0 | 395.5126877834758 |
| 12 | 282.0 | 247.45232434195054 |
| 13 | 222.0 | 304.4541168026751 |
| 14 | 153.0 | 290.62845417789504 |
| 15 | 102.0 | 32.645238554847424 |
| 16 | 131.0 | 77.99562938208169 |
| 17 | 117.0 | 89.85895463164297 |
| 18 | 84.0 | 77.62906961944464 |
| 19 | 110.0 | 11.591000000000008 |
| 20 | 77.0 | 20.294505403013602 |
| 21 | 55.0 | 31.062630289576703 |
| 22 | 27.0 | 6.736759218026009 |
| 23 | 20.0 | 11.511796502496054 |
| 24 | 6.0 | 87.91855885502832 |
| 25 | 3.0 | -40.91229918336927 |
| 26 | 2.0 | -46.40862039104417 |
| 27 | 1.0 | 91.41625472239264 |
| 28 | 2.0 | 48.21443801035133 |
| 29 | 2.0 | 180.88182388820485 |
| 30 | 2.0 | -14.52992543117466 |
| 31 | 3.0 | 134.4563535426497 |
| 32 | 0.0 | -2.7547178918988493 |
| 33 | 1.0 | 285.94510690391496 |
| 34 | 5.0 | -49.574082652826974 |
| 35 | 2.0 | -145.30589128109625 |
| 36 | 1.0 | -205.50464464236128 |
| 37 | 2.0 | 4.344305040016025 |
| 38 | 4.0 | 11.954325249446697 |
| 39 | 1.0 | -6.0350835603212545 |
| 40 | 5.0 | 27.170621298309925 |
| 41 | 2.0 | -108.26805749401524 |
| 42 | 4.0 | -28.251340509725367 |
| 43 | 3.0 | -49.96319854812282 |
| 44 | 6.0 | 3.1269092634009894 |
| 45 | 10.0 | -46.51183287970025 |
| 46 | 10.0 | -35.62794069122435 |
| 47 | 11.0 | -157.06562443293012 |
| 48 | 28.0 | -18.400123979183036 |
| 49 | 36.0 | 1.1580673925479914 |
| 50 | 37.0 | -30.30035354287844 |
| 51 | 60.0 | 142.97320440476915 |
| 52 | 134.0 | 226.15626412556594 |
| 1 | 278.0 | 707.8169725304942 |
| 2 | 598.0 | 695.0152560417905 |
| 3 | 1141.0 | 1281.0287767049676 |
| 4 | 1813.0 | 1832.2992439161303 |
| 5 | 2051.0 | 1865.3817475042683 |
| 6 | 1521.0 | 1400.8642722093332 |
| 7 | 1378.0 | 1434.2655732075777 |
| 8 | 1098.0 | 1030.6891199370632 |
| 9 | 1135.0 | 893.4063454588736 |
| 10 | 875.0 | 675.1290040417693 |
| 11 | 765.0 | 302.22268778347575 |
| 12 | 758.0 | 434.0323243419506 |
| 13 | 694.0 | 397.7441168026751 |
| 14 | 479.0 | 234.654454177895 |
| 15 | 359.0 | 191.2382385548475 |
| 16 | 272.0 | 77.99562938208169 |
| 17 | 205.0 | 33.884954631642984 |
| 18 | 128.0 | -52.9769303805553 |
| 19 | 69.0 | 86.22299999999996 |
| 20 | 44.0 | 10.965505403013594 |
| 21 | 31.0 | 12.404630289576744 |
| 22 | 18.0 | 90.69775921802602 |
| 23 | 10.0 | -44.46220349750399 |
| 24 | 5.0 | -154.6354411449717 |
| 25 | 7.0 | -143.5312991833693 |
| 26 | 5.0 | -93.05362039104415 |
| 27 | 5.0 | -95.16374527760729 |
| 28 | 3.0 | -110.37856198964869 |
| 29 | 6.0 | -332.2131761117951 |
| 30 | 6.0 | -173.12292543117468 |
| 31 | 3.0 | -126.75564645735028 |
| 32 | 3.0 | -217.32171789189894 |
| 33 | 5.0 | -124.53089309608515 |
| 34 | 3.0 | -49.574082652826974 |
| 35 | 4.0 | -98.66089128109621 |
| 36 | 3.0 | 93.0233553576387 |
| 37 | 3.0 | -98.274694959984 |
| 38 | 3.0 | -81.33567475055332 |
| 39 | 3.0 | -108.65408356032128 |
| 40 | 5.0 | -28.803378701690065 |
| 41 | 5.0 | -61.62305749401526 |
| 42 | 2.0 | -18.92234050972536 |
| 43 | 7.0 | -87.27919854812279 |
| 44 | 7.0 | 96.41690926340107 |
| 45 | 17.0 | 65.43616712029979 |
| 46 | 19.0 | 11.017059308775629 |
| 47 | 40.0 | -82.43362443293012 |
| 48 | 83.0 | 121.53487602081702 |
| 49 | 126.0 | 94.44806739254796 |
| 50 | 295.0 | 221.58264645712154 |
| 51 | 752.0 | 469.48820440476925 |
| 52 | 1947.0 | 1028.4502641255658 |
| 1 | 2669.0 | 2844.1579725304946 |
| 2 | 2985.0 | 1739.8632560417905 |
| 3 | 2243.0 | 1523.5827767049677 |
| 4 | 1742.0 | 1711.0222439161303 |
| 5 | 1176.0 | 1762.7627475042682 |
| 6 | 733.0 | 1391.535272209333 |
| 7 | 489.0 | 1210.3695732075778 |
| 8 | 325.0 | 769.4771199370632 |
| 9 | 305.0 | 734.8133454588735 |
| 10 | 208.0 | 535.1940040417694 |
| 11 | 209.0 | 442.1576877834758 |
| 12 | 197.0 | 378.05832434195054 |
| 13 | 152.0 | 407.07311680267514 |
| 14 | 181.0 | 178.68045417789506 |
| 15 | 115.0 | 191.2382385548475 |
| 16 | 111.0 | 236.58862938208176 |
| 17 | 103.0 | 80.52995463164302 |
| 18 | 91.0 | -80.96393038055533 |
| 19 | 54.0 | 384.751 |
| 20 | 28.0 | 20.294505403013602 |
| 21 | 15.0 | 3.0756302895767362 |
| 22 | 14.0 | 16.065759218026017 |
| 23 | 13.0 | 104.80179650249602 |
| 24 | 7.0 | 78.58955885502837 |
| 25 | 4.0 | 127.0097008166307 |
| 26 | 4.0 | 18.89437960895583 |
| 27 | 4.0 | 44.77125472239271 |
| 28 | 5.0 | -119.7075619896487 |
| 29 | 5.0 | -61.672176111795125 |
| 30 | 7.0 | 97.41807456882532 |
| 31 | 5.0 | 181.1013535426497 |
| 32 | 3.0 | 407.72128210810115 |
| 33 | 4.0 | 80.7071069039149 |
| 34 | 4.0 | 15.728917347173024 |
| 35 | 5.0 | 190.5381087189038 |
| 36 | 3.0 | 148.99735535763875 |
| 37 | 4.0 | -60.95869495998397 |
| 38 | 4.0 | -146.63867475055332 |
| 39 | 3.0 | 460.4149164396788 |
| 40 | 4.0 | 129.78962129830995 |
| 41 | 6.0 | 59.65394250598479 |
| 42 | 5.0 | 18.393659490274615 |
| 43 | 10.0 | 117.9588014518772 |
| 44 | 5.0 | -108.82109073659899 |
| 45 | 4.0 | 168.05516712029976 |
| 46 | 13.0 | -7.640940691224273 |
| 47 | 11.0 | 29.514375567069862 |
| 48 | 7.0 | 261.46987602081697 |
| 49 | 13.0 | 1.1580673925479914 |
| 50 | 17.0 | 35.00264645712156 |
| 51 | 29.0 | 142.97320440476915 |
| 52 | 66.0 | 142.19526412556593 |
| 53 | 120.0 | 120.03186653396358 |
| 1 | 178.0 | -131.79302746950577 |
| 2 | 284.0 | 1012.2012560417907 |
| 3 | 510.0 | 1141.0937767049677 |
| 4 | 978.0 | 1608.4032439161301 |
| 5 | 1665.0 | 1884.0397475042682 |
| 6 | 2472.0 | 2147.1842722093334 |
| 7 | 2343.0 | 1592.8585732075778 |
| 8 | 2072.0 | 1515.7971199370634 |
| 9 | 2021.0 | 1285.2243454588736 |
| 10 | 1460.0 | 908.3540040417695 |
| 11 | 1043.0 | 880.6206877834757 |
| 12 | 813.0 | 629.9413243419506 |
| 13 | 755.0 | 593.6531168026752 |
| 14 | 449.0 | 439.89245417789505 |
| 15 | 188.0 | 321.8442385548475 |
| 16 | 154.0 | 189.94362938208178 |
| 17 | 86.0 | 164.49095463164304 |
| 18 | 43.0 | 180.24806961944478 |
| 19 | 22.0 | -53.71199999999999 |
| 20 | 18.0 | 29.62350540301361 |
| 21 | 9.0 | -43.5693697104233 |
| 22 | 5.0 | -21.250240781973957 |
| 23 | 5.0 | 39.49879650249602 |
| 24 | 3.0 | 59.93155885502836 |
| 25 | 2.0 | -12.925299183369305 |
| 26 | 3.0 | 205.47437960895581 |
| 27 | 3.0 | 54.10025472239272 |
| 28 | 4.0 | 85.5304380103513 |
| 29 | 5.0 | -71.00117611179508 |
| 30 | 5.0 | 125.4050745688254 |
| 31 | 3.0 | -61.45264645735028 |
| 32 | 11.0 | 155.8382821081011 |
| 33 | 3.0 | -143.18889309608517 |
| 34 | 2.0 | 127.676917347173 |
| 35 | 6.0 | 69.26110871890376 |
| 36 | 1.0 | 121.01035535763867 |
| 37 | 1.0 | 153.60830504001603 |
| 38 | 7.0 | 385.11432524944667 |
| 39 | 8.0 | 115.24191643967879 |
| 40 | 15.0 | -28.803378701690065 |
| 41 | 17.0 | 171.60194250598477 |
| 42 | 16.0 | 102.35465949027457 |
| 43 | 25.0 | 61.98480145187716 |
| 44 | 38.0 | 105.74590926340102 |
| 45 | 52.0 | 37.44916712029976 |
| 46 | 79.0 | 76.32005930877563 |
| 47 | 93.0 | 188.10737556706988 |
| 48 | 122.0 | 28.24487602081706 |
| 49 | 170.0 | 225.05406739254795 |
| 50 | 203.0 | 380.1756464571216 |
| 51 | 422.0 | 460.1592044047692 |
| 52 | 740.0 | 1037.779264125566 |
| 1 | 1076.0 | 997.0159725304943 |
| 2 | 1308.0 | 1124.1492560417905 |
| 3 | 1701.0 | 1346.3317767049678 |
| 4 | 2393.0 | 1673.70624391613 |
| 5 | 2562.0 | 2070.619747504268 |
| 6 | 1786.0 | 1699.392272209333 |
| 7 | 1451.0 | 1294.3305732075778 |
| 8 | 906.0 | 974.7151199370633 |
| 9 | 608.0 | 837.4323454588734 |
| 10 | 525.0 | 647.1420040417695 |
| 11 | 379.0 | 507.4606877834758 |
| 12 | 295.0 | 490.0063243419505 |
| 13 | 239.0 | 369.7571168026751 |
| 14 | 218.0 | 281.2994541778951 |
| 15 | 157.0 | 209.8962385548475 |
| 16 | 97.0 | 124.64062938208167 |
| 17 | 98.0 | 155.16195463164308 |
| 18 | 51.0 | 273.53806961944474 |
| 19 | 52.0 | -25.725000000000023 |
| 20 | 34.0 | 48.281505403013625 |
| 21 | 30.0 | 77.70763028957674 |
| 22 | 16.0 | 137.342759218026 |
| 23 | 16.0 | 20.840796502496005 |
| 24 | 8.0 | 78.58955885502837 |
| 25 | 6.0 | 80.36470081663072 |
| 26 | 5.0 | 0.236379608955815 |
| 27 | 7.0 | -1.873745277607327 |
| 28 | 11.0 | 160.16243801035125 |
| 29 | 12.0 | 236.8558238882049 |
| 30 | 9.0 | 4.128074568825355 |
| 31 | 7.0 | -24.136646457350253 |
| 32 | 8.0 | -58.728717891898896 |
| 33 | 9.0 | -96.54389309608507 |
| 34 | 13.0 | 15.728917347173024 |
| 35 | 14.0 | -61.34489128109624 |
| 36 | 7.0 | -28.25364464236128 |
| 37 | 15.0 | 69.64730504001602 |
| 38 | 6.0 | -25.361674750553334 |
| 39 | 12.0 | -183.28608356032123 |
| 40 | 19.0 | -66.1193787016901 |
| 41 | 13.0 | 78.3119425059848 |
| 42 | 14.0 | -65.5673405097254 |
| 43 | 15.0 | 52.65580145187721 |
| 44 | 19.0 | -34.189090736598985 |
| 45 | 29.0 | -9.19583287970022 |
| 46 | 23.0 | 66.99105930877568 |
| 47 | 34.0 | 216.09437556706985 |
| 48 | 78.0 | -9.071123979182971 |
| 49 | 106.0 | 122.43506739254792 |
| 50 | 226.0 | 118.96364645712151 |
| 51 | 460.0 | 385.52720440476924 |
| 52 | 785.0 | 1075.0952641255658 |
